# KITSUNE: A Tool for Identifying Empirically Optimal K-mer Length for Alignment-Free Phylogenomic Analysis

**DOI:** 10.3389/fbioe.2020.556413

**Published:** 2020-09-23

**Authors:** Natapol Pornputtapong, Daniel A. Acheampong, Preecha Patumcharoenpol, Piroon Jenjaroenpun, Thidathip Wongsurawat, Se-Ran Jun, Suganya Yongkiettrakul, Nipa Chokesajjawatee, Intawat Nookaew

**Affiliations:** ^1^Department of Biochemistry and Microbiology, Faculty of Pharmaceutical Sciences, and Research Unit of DNA Barcoding of Thai Medicinal Plants, Chulalongkorn University, Bangkok, Thailand; ^2^Department of Biomedical Informatics, University of Arkansas for Medical Sciences, Little Rock, AR, United States; ^3^Joint Graduate Program in Bioinformatics, University of Arkansas at Little Rock and University of Arkansas for Medical Sciences, Little Rock, AR, United States; ^4^National Center for Genetic Engineering and Biotechnology, National Science and Technology Development Agency, Pathum Thani, Thailand

**Keywords:** k-mer, species identification, phylogenomics, comparative genomics, alignment-free

## Abstract

Genomic DNA is the best “unique identifier” for organisms. Alignment-free phylogenomic analysis, simple, fast, and efficient method to compare genome sequences, relies on looking at the distribution of small DNA sequence of a particular length, referred to as k-mer. The k-mer approach has been explored as a basis for sequence analysis applications, including assembly, phylogenetic tree inference, and classification. Although this approach is not novel, selecting the appropriate k-mer length to obtain the optimal resolution is rather arbitrary. However, it is a very important parameter for achieving the appropriate resolution for genome/sequence distances to infer biologically meaningful phylogenetic relationships. Thus, there is a need for a systematic approach to identify the appropriate k-mer from whole-genome sequences. We present K-mer–length Iterative Selection for UNbiased Ecophylogenomics (KITSUNE), a tool for assessing the empirically optimal k-mer length of any given set of genomes of interest for phylogenomic analysis via a three-step approach based on (1) cumulative relative entropy (CRE), (2) average number of common features (ACF), and (3) observed common features (OCF). Using KITSUNE, we demonstrated the feasibility and reliability of these measurements to obtain empirically optimal k-mer lengths of 11, 17, and ∼34 from large genome datasets of viruses, bacteria, and fungi, respectively. Moreover, we demonstrated a feature of KITSUNE for accurate species identification for the two *de novo* assembled bacterial genomes derived from error-prone long-reads sequences, and for a published yeast genome. In addition, KITSUNE was used to identify the shortest species-specific k-mer accurately identifying viruses. KITSUNE is freely available at https://github.com/natapol/kitsune.

## Introduction

Genome sequences have been used widely for species identification with high accuracy and have been useful to many research areas in the biotechnological (Costessi et al., 2018), environmental (Vandenkoornhuyse et al., 2010), evolutionary (Bruger and Marx, 2018; Sands, 2019), and clinical sciences (Balloux et al., 2018). With the rapid technological development of genome sequencing, more and more organisms have been sequenced across all kingdoms (Galagan et al., 2005; Land et al., 2015; Houldcroft et al., 2017; Chen et al., 2019; Rexroad et al., 2019). The enormous amount of data generated by sequencing has made it challenging to compare sequences with alignment-based approaches such as BLAST (Altschul et al., 1990). The alignment-based approach generally requires significant memory and is time consuming, making the comparison of multi-genome-scale sequence data infeasible. Therefore, alignment-free methods for biological sequence analysis have been developed and perform well for comparative genomics and metagenomics, while also being less time consuming than alignment-based methods (Ren et al., 2018).

The alignment-free approach, which is simple, efficient, and fast, relies on looking at the distribution of small consecutive pieces of DNA sequences, called k-mers. The k-mer–based approach has been applied to several types of biological sequence analyses, including assembly (Sohn and Nam, 2018), phylogenetic tree inference (Bernard et al., 2016, 2019; Thankachan et al., 2017; Zhang et al., 2017; Tang et al., 2019; Choi and Kim, 2020), and microbial/microbiome classification (Brinda et al., 2015; Ondov et al., 2016; Lu et al., 2017; Jain et al., 2018; Tang et al., 2019; Wood et al., 2019). A detailed assessment of different k-mer-counting algorithms was reported by Manekar and Sathe (2018), and rigorous comparisons and benchmarking of different alignment-free methods were provided in published reviews (Bonham-Carter et al., 2014; Zielezinski et al., 2017, 2019). Although the k-mer–based approach is not novel, selecting the appropriate k-mer length to obtain the good resolution in specific applications can be arbitrary. Nevertheless, k-mer length is a very important parameter in alignment-free phylogenetic inference (Bernard et al., 2019).

Empirically optimal k-mer is defined as the k-mer length that give a good discrimination among a considered set of genomes. A previous study attempted to calculate empirically optimal k-mer length based on cumulative relation entropy (CRE) and relative sequence divergence (Wu et al., 2009), which provided a foundation to choose the empirically optimal k-mer length. Bai et al. (2017) proposed a theoretical framework to define the empirically optimal k-mer length based on Markov chains modeling and the Chi-square statistic. However, we proposed a three-step approach based on information content (Zhang et al., 2017) to more systematically assess the empirically optimal k-mer length. Our approach produced a successful alignment-free phylogenomic analysis of thousands of viral genomes (Zhang et al., 2017). Here, we present KITSUNE (K-mer–length Iterative Selection for UNbiased Ecophylogenomics) software for identifying the empirically optimal k-mer length from a given set of genomes for phylogenomic analysis. The “empirically optimal k-mer length” could be defined as a selected k-mer length that gives a well distributed genomic distances that can be used to infer biologically meaningful phylogenetic relationships. In addition, the software provides various genomic distance estimations based on the k-mer frequency profile that can be used for inferring phylogenomic trees, identifying species, and identifying unique species-specific sequences for use as genetic markers.

## Methods

### Software Implementation

K-mer–length Iterative Selection for UNbiased Ecophylogenomics was implemented in Python programming language version 3.6. KITSUNE first uses Jellyfish software (Marcais and Kingsford, 2011) to generate a k-mer frequency profile from a FASTA file and stores the k-mer profile in sparse matrix format. This is a suitable representation because k-mer profiles are usually sparse due to the very large number of possible k-mers (<4^*k*^), and this representation still allows for an efficient calculation.

#### The Three-Step Approach to Identify Empirically Optimal K-mer Length

**FIGURE 1:**
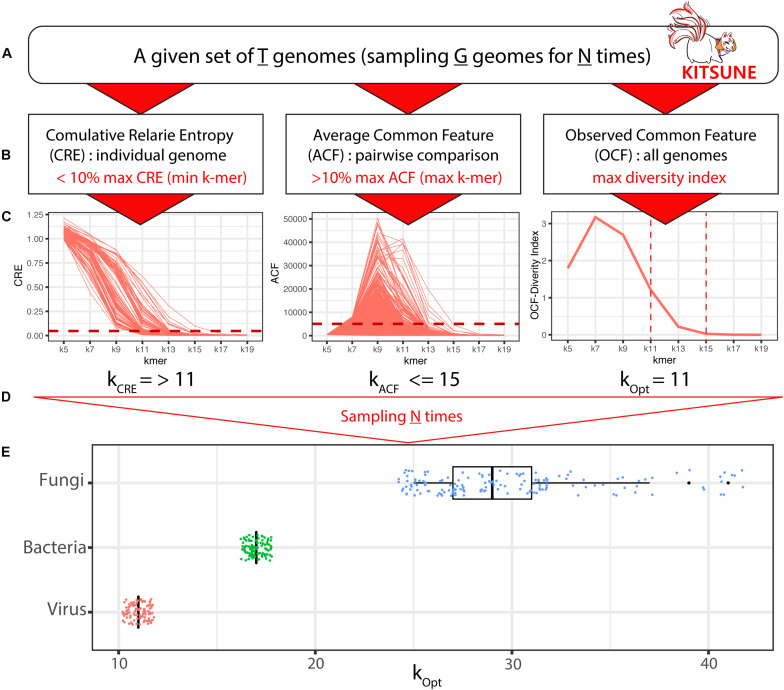
A 3-step approach for identifying empirically optimal k-mer length with KITSUNE. The KITSUNE begins with the following: **(A)** select a given set of genomes (T) that will be used for subsampling genomes (G) from T for N times and **(B)** apply the three-step approach at the desired range of k-mer lengths using CRE to obtain the lower bound of k-mer length (using the <10% of maximum CRE as the cut-off), using ACF to obtain the upper bound of k-mer length (using the >10% of maximum ACF as the cut-off), and using OCF to select the k-mer lengths that have the highest diversity within the identified bounds. **(C)** Illustration of ACF, CRE, and OCF for a single sampling. **(D)** The final step is to repeat the calculation for different samplings N times. **(E)** Empirically optimal k-mer lengths based on individual samplings (dots) and mean values (boxplots) for viral (red), bacterial (green), and fungal (blue) genome datasets.

This k-mer frequency profile enables users to calculate three values, which are used for the three-step approach (Zhang et al., 2017) as summarized in Figure 1. KITSUNE provides three key commands, which are “*cre*,” “*acf*,” and “*ocf*,” to calculate cumulative relative entropy (CRE), average number of common features (ACF), and observed common features (OCF), respectively. The formulas for CRE, ACF, and OCF are as follows:

1. CRE:

(1) $$\text{CRE}\,\left(l\right)=\sum_{k=l}^{\infty }\text{RE}\,\left(F_{k},{\hat{F}}_{k}\right)$$

(2) $$\text{RE}\,\left(F_{l},{\hat{F}}_{k}\right)=\sum_{i}f_{i,l}\,{\text{log}}_{2}\,\frac{f_{i,l}}{{\hat{f}}_{i,k}}$$

Here, *l* is the feature (*k-mer*) length, *f*_*i,l*_ is the observed feature frequency of feature *i* of length *l*, and ${\hat{f}}_{i,l}$ is the expected feature frequency formulated from the Markov model of feature *i*, as described in a previous study (Wu et al., 2009). The CRE value indicates the amount of information from the genome sequence encoded in a k-mer profile; it represents the relative information content of an individual genome over k-mer lengths. The CRE decreases monotonically as k-mer length increases (Figure 1C). The k-mer lengths that give a CRE of close to zero for an individual genome are selected to infer approximate information for increasingly longer k-mer lengths (Wu et al., 2009).

2. ACF:

(3) $$\text{ACF}\,\left(l\right)=\sum_{j\neq i}\frac{c\,\left(g_{i},g_{j},l\right)}{\left(T-1\right)}$$

Average number of common features is calculated as an ACF between one to all genomes where *c*(*g*_*i*_,*g*_*j*_,*l*) is a number of common k-mers of length *l* between genome *g*_*i*_ and genome *g*_*j*_, and *T* is the number of genomes in the dataset. ACF represents the commonality between 2 genomes. The k-mer length that gives a value of zero value for ACF will produce a random relationship. In contrast, very high ACF will give poor discrimination because most of features are in common (Zhang et al., 2017). Therefore, the k-mer lengths that do not give ACF = 0 are selected.

3. OCF:

Observed common features includes unique and non-unique k-mers based on the occurrence of k-mers among all considered genomes at a specific k-mer length. The probability of features being found in individual genomes ($p_{i,}\,\sum_{i=1}^{T}p_{i}=1$) is calculated and used as the input for calculating the Shannon diversity index (*H*) to estimate the level of similarity and dissimilarity across all considered genomes, where *i* represents the individual genome and *N* is the total number of considered genomes. *p*_*i*_ can be calculated as C_*i*_/O_*k*_, where O_*k*_ (O_*k*_ ≤ 4^*k*^) is the number of all observed k-mers for a specific k-mer length k, and C_*i*_ are the number of k-mers found in i genome (1 ≤ i ≤ T), see Zhang et al. (2017) for example of the calculation.

(4) $$H=-\sum_{i=1}^{T}p_{i}\,l\,n\,p_{i}$$

*H* gives the degree of commonness of k-mers among all consider genomes, therefore a higher value for *H* is preferred to keep higher diversity in the commonness of k-mers.

Further detail on this three-step approach is available in our previous study (Zhang et al., 2017). In most cases, an empirically optimal k-mer length would give CRE and ACF values that are 10% of their maximum values and where the k-mer is not unique to a genome. CRE and ACF will give a minimum and maximum boundary for the k-mer length. Then, the Shannon diversity index based on OCF will be used to select the k-mer length in the range obtained from ACF and CRE that has the highest diversity (*H*). The k-mer selected based on the three-step approach gives the optimal distance among the viral genomes that can be used to delineate biologically meaningful phylogenetic relationships (Zhang et al., 2017). Each of these measurements gives the user a quantitative value to guide them on which k-mer length should be selected for further analysis. The CRE, ACF, and OCF complement each other in selecting the empirically optimal k-mer in a dataset of interest because they measure an empirically optimal range of k-mers from a different perspective.

#### Genomic Distance Estimation

K-mer–length Iterative Selection for UNbiased Ecophylogenomics provides 18 methods for calculating the genomic distance based on standard dissimilarity: i.e., Bray–Curtis, Canberra, Chebyshev, City Block (Manhattan), Correlation, Cosine, Euclidean, Jensen–Shannon, Squared Euclidean, Dice, Hamming, Jaccard, Kulsinski, Rogers–Tanimoto, Russell–Rao, Sokal–Michener, Sokal–Sneath, and Yule. KITSUNE also provides the transformation distance based on the formula presented by Fan et al. (2015), which is used for Mash (Ondov et al., 2016) and FastANI (Jain et al., 2018) for genomic distance calculation, that is

(5) $$\mathrm{Transformation}\,\mathrm{distance}=-\frac{1}{k}\,\ln\left(\frac{2\,j}{1+j}\right)$$

where k is the considered k-mer length and j is the similarity index (1 – distance) between two genomes. The transformed genomic distances can be used for species identification.

#### Overview of KITSUNE Features and Uses

The input files for KITSUNE are the genome sequences of the organism of interest in standard FASTA format. KITSUNE provides three core functions, “*cre,” “acf,” and “ocf”* to calculate the CRE, ACF, and OCF, respectively, which are the three matrices for empirically optimal k-mer length identification, at a given k-mer length. The three functions are used as the basis to identify the empirically optimal k-mer length of a given genome sequences, based on the proposed three-step approach in the wrap-up function *“kopt.”* Users can specify the largest k-mer length for the “*kopt*” function, and the empirically optimal k-mer length will be reported if available; users can change the cut-off values for the two matrices (CRE and ACF). In addition, KITSUNE provides the function *“dmatrix”* for genomic distance calculations reported in the matrix format with the option of many distances method with or without transformation (Eq. 5). These functions provide a convenient analysis of empirically optimal k-mer length coupling the genomic distance calculations in the same package. Lastly, because “*acf*” calculates the ACF between genome sequences, the user can apply the function to identify a unique k-mer for an organism of interest. KITSUNE is designed to analyze assembled genomes, not data from deep sequencing; however, the identified empirically optimal k-mer length could guide the analysis of deep-sequencing data using other alignment-free tools.

### Publicly Available Datasets Used in the Study

#### Genome Datasets

We downloaded nucleotide FASTA files for 8,967 viral reference genomes and 8,861 complete bacterial genomes from the National Center for Biotechnology Information (NCBI) database and 729 fungal genomes from the JGI MycoCosm database (Grigoriev et al., 2014). These datasets were used to identify empirically optimal k-mer lengths and to identify species in each domain. All of genomes in the datasets is listed in the Supplementary Table 1.

#### Yeast Genome for Evaluating the Identification of Fungal Species Identification at Different k-mer Lengths

We used the well-characterized *Saccharomyces cerevisiae* genome strain CEN.PK113-7D genome (Jenjaroenpun et al., 2018) to evaluate the ability of KITSUNE to identify species using different k-mer lengths. The yeast genome was downloaded from NCBI under BioProject accession number PRJNA398797.

#### *Escherichia coli-Shigella* Dataset for Evaluating the Identification of Closely Related Species

*Escherichia coli* reference genomes from known five phylogroups, which are A, B1, B2, D, and E (Gordon et al., 2008; Skippington and Ragan, 2011), and *Shigella* sp. genomes that were used previously for phylogenetic analysis (Bernard et al., 2016; Lu et al., 2017) were downloaded from the NCBI database. Only chromosomal genome sequences were used for the analysis. All of genomes in the datasets is listed in the Supplementary Table 1.

#### Selected Viral Genomes for Evaluating the Identification of Shortest Species-Specific Sequence

We arbitrarily selected and downloaded seven complete viral genomes associated with human diseases from different taxonomic ranks from NCBI: Chikungunya virus (CHIKV), Dengue virus (DENV) (Kinney et al., 1997), human immunodeficiency virus 1 (HIV) (Martoglio et al., 1997), influenza A virus (IAV) (Fields and Winter, 1982), Zika virus (ZIKV) (Wongsurawat et al., 2018a,b), severe acute respiratory syndrome coronavirus 2 (SARS-CoV-2) (Harcourt et al., 2020), and Kaposi’s sarcoma-associated human herpes virus (KSHV) strain GK18 (NC_009333) (Rezaee et al., 2006) (a DNA virus that can interact with the host through an RNA/DNA hybrid mechanism) (Wongsurawat et al., 2020).

### Bacterial Culture and DNA Purification

*Lactobacillus plantarum* BCC9546 (BIOTEC culture collection, Thailand) (Chokesajjawatee et al., 2020) was grown in (Man, Rogosa, and Sharpe) MRS broth at 30°C for 16 h. The genomic DNA was extracted with the Wizard Genomic DNA Purification Kit (Promega, United States).

*Streptococcus suis* HU_SS30, isolated from a human patient in Thailand, was grown in 10 mL of Todd Hewitt broth (Oxoid Limited, Hampshire, United Kingdom) containing 0.2% yeast extract at 37°C with 5% CO_2_ for 18 h. The genomic DNA was extracted with the Quick-DNA^TM^ Fungal/Bacterial Microprep Kit (Zymo Research, Irvine, CA, United States). The study was carried out in strict compliance with the recommendations and approval of the ethical committee of Thammasat University (Protocol Number 10/2557) and the biosafety committee of Thammasat University (protocol number 021/2557 and 036/2561).

### Whole Genome Sequencing Using the Oxford Nanopore Technologies Platform

Approximately 400 ng of purified DNA from each bacterial species was used as the input for the Rapid Barcoding Kit RBK004 (ONT, United States) to prepare the sequencing library. The library was loaded into an R9.4/FLOMIN106 flow cell in a MinION sequencing device. MinKNOW software was used to control the sequencing run and data acquisition for 48 h. The raw fastq data was deposited in the Sequence Read Archive database under BioProject number PRJNA644942

### ONT Data Processing and *de novo* Genome Assembly

The raw Oxford Nanopore Technologies (ONT) signals were base-called using Albacore v2.3.4 (ONT) to generate FASTQ reads, and adapter sequences were trimmed with Porechop v0.2.3 using default parameters. Genome assembly was performed on reads that were longer than 200 bp. Flye assembler (Kolmogorov et al., 2019) version 2.5 was applied to the assembled genomes using default parameters to obtain the complete chromosomes and plasmids. The assembled contigs were visualized and plotted using Bandage software version 0.8.1 (Wick et al., 2015).

## Results

### Identification of Empirically Optimal K-mer Lengths for Viral, Bacterial, and Fungal Genomes Using KITSUNE

K-mer–length Iterative Selection for UNbiased Ecophylogenomics provides three matrices—CRE, ACF, and OCF—to identify the empirically optimal k-mer length for a given set of genomes (T) for making alignment-free comparisons between genomes (Figure 1). The empirically optimal k-mer length was calculated based on our three-step approach (Zhang et al., 2017; see Figure 1B): step (1) we selected k-mers length that gave CRE < 10% of the maximum to define the lower bound of k-mer length (minimum k); step (2) we selected k-mers length that gave ACF > 10% of the maximum to define the upper bound of k-mer length (maximum k); and step (3) we selected k-mer length within the minimum and the maximum of k-mer length that yield the highest diversity index (*H*) based on OCF.

We applied the KITSUNE workflow (see Figure 1) to identify the empirically optimal k-mer length for our selected reference viral genomes (T = 8,967 genomes), complete bacterial genomes (T = 8,861 genomes), and fungal genomes (T = 729 genomes). Identifying the empirically optimal k-mer length requires many iterations over different k-mer lengths; therefore, using all genomes in the iterative calculations requires significant computational resources. Instead, we used a random sampling approach to perform the iterative calculations across considered k-mer lengths on subsets of all genomes/subsample (G genomes) several times (N times). For the viral and bacterial genome datasets, we sampled 100 genomes (G = 100) 100 times (N = 100). For the fungal genome dataset, we sampled eight genomes (G = 8) 300 times (N = 300); this was due to the larger genome size of fungi and our available computational resources (RAM ∼ 200 GB). For the example of an iteration shown in Figure 1C, CRE gave a minimum k-mer length of 11 and ACF gave a maximum k-mer length of 15; a k-mer length of 11 was selected because it gave the highest diversity (*H*) within the k-mer length range of 11 and 15. The results for empirically optimal k-mer length derived from the iterative calculation are summarized in the boxplots in Figure 1E. We identified the empirically optimal k-mer length to be 11 for the viral genome dataset and 17 for the bacterial genome dataset.

Interestingly, the empirically optimal k-mer length, identified with individual sampling (subsample), was the same value for the viral and bacterial datasets. On the other hand, the empirically optimal k-mer length obtained from individual sampling of the fungal genome dataset varied from 23 to 43, with a mean of 37, indicating the insufficient sample size (G = 8). The subsample size is an important factor; therefore, we evaluated the impact of subsample size for the viral genome dataset. We found that a subsample of 20 viral genomes was sufficient to achieve convergence from individual iterations and gave the same empirically optimal k-mer length of 11 as subsample of 100 viral genomes (Supplementary Figure S1).

### Comparison of Genomic Distances

**FIGURE 2:**
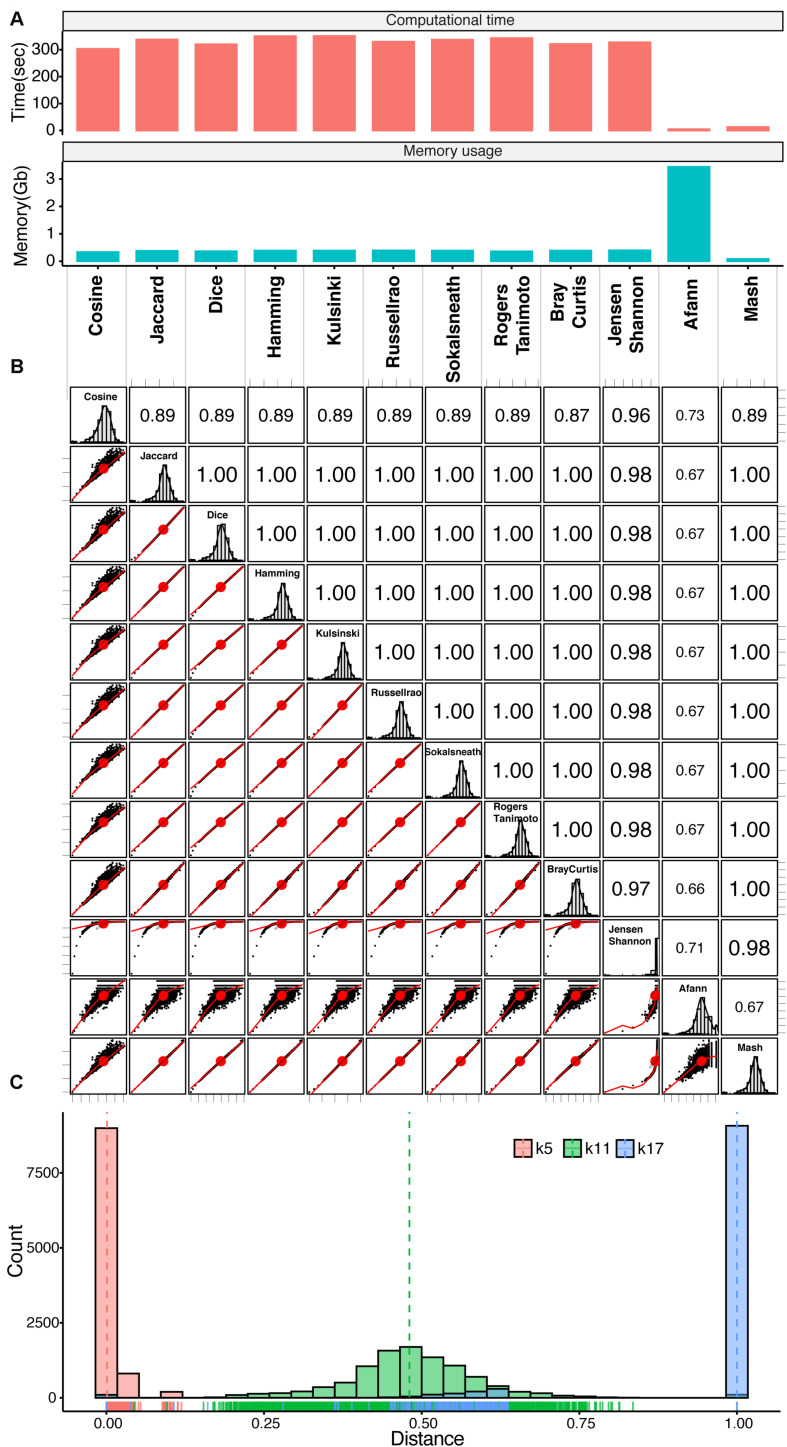
Genomic distance comparison. **(A)** Bar plots of computational time and memory required for genomic distance calculation by individual methods. **(B)** Correlogram plot comparing transformed genomic distances calculated with different methods based on 100 viral genomes. Lower left triangle: scatter plots of genomic distances derived from of pairs of genomes calculated with different methods with correlation ellipses; upper right triangle: Spearman’s rank correlation coefficients for different methods; diagonal boxes: distribution of genomic distances. **(C)** Histograms with rug plots show the distributions of genomic distances (transformed Jaccard) based on the identified empirically optimal k-mer length (k11, green), k-mer length of 5 (k5, red), and k-mer length of 17 (k17, blue). See Supplementary Figure S2 for the distribution of genomic distances derived from all k-mer-length from 5 to 17.

We next compared different methods for calculating genomic distances using our empirically optimal viral k-mer length of 11, derived from 100 viral genomes as an example. We arbitrarily selected 11 methods, computed genomic distances, and compared them as shown in Figure 2. We also included the Mash method (Ondov et al., 2016) and the Afann method (Tang et al., 2019) in the comparison. Mash uses the MinHash/Sketching algorithm (Broder, 1977; Indyk and Motwani, 1998) to reduce the data size of k-mer frequency profiles and calculates the distance by a transformation of the Jaccard index, which is calculated from MinHash based on the formula presented by Fan et al. (2015) (Eq. 5). Afann uses neural network regression to adjust the sequence biases and then calculates genomic distance using background adjustment methods such as d2S and d2Star (Reinert et al., 2009). The computational time and memory use of different methods, including Mash, and Afann (with the 100 viral genomes), were compared, as illustrated in Figure 2A. As expected, the Mash method had the second shortest computational time and lowest memory use because the data were compressed by the MinHash/Sketching algorithm (Broder, 1977; Indyk and Motwani, 1998). The computational time for the d2Star distance derived from Afann was the shortest, but Afann used more memory than the others. The distances derived from KITSUNE required similar computational times, which were longer than Afann and Mash software.

We next computed the genomic distances of the 100 viral genomes using the standard distance formula for the 10 individual methods and compared them with the genomic distances calculated with Mash (Supplementary Figure S3). The scatter plots for all of the 10 distances had clear linear relationships with each other but not with the Mash distance. Unlike Mash distances, which have a normal distribution (see diagonal boxes), the 10 distances had a strong right-hand skew close to 1. The d2Star distances derived from Afann had a sharp distribution of around 0.5. However, all of them had a good correlation based on rank (Spearman’s rank correlation coefficient >0.69). We then applied the transformation formula (Eq. 5) presented by Fan et al. (2015) to calculate distances for all 11 methods and d2star then compared them together with the Mash distance (Figure 2B). After transformation, there was a clear linear relationship among the distances calculated with almost every method (see scatter plots), each with a normal distribution (see diagonal boxes), except for the Jensen–Shannon method. Nevertheless, the transformation did not much change the rank correlation among them (Spearman’s correlation coefficient >0.66).

We next determined the impact of k-mer lengths on genomic distance (transformed Jaccard) as illustrated in the histogram plot of Figure 2C for the identified empirically optimal k-mer length of 11 with k-mer length of 5 and 17 (see Supplementary Figure S2 for other k-mer lengths and a tree shows good discrimination among different virus families/genus using the empirically optimal k-mer length of 11). At k-mer length of 5, which is too short, most of the genomic distances were close to 0; therefore, discrimination among the genomes was limited. On the other hand, at a k-mer length is 17, which is too long, most of the genomic distances were close to 1; therefore, discrimination among the genomes was saturated. At the empirically optimal k-mer length of 11, the distribution of genomic distances was normally distributed near in the middle of the distance scale. This characteristic could be used to infer biologically meaningful phylogenetic relationships that need further investigations.

### *De novo* Assembly of Bacterial Genomes From ONT Sequencing for Species Identification

ONT provides long-read sequencing (>10 kb), which overcomes the issue of assembling disambiguated reads from short-read sequencing data. This allows users to obtain contiguous chromosomal and plasmid sequences (Jenjaroenpun et al., 2018; De Maio et al., 2019). Unfortunately, because of the higher sequencing error rate of ONT over short-read sequencing, approximately 1% of errors remain in the assembled sequence even after self-consensus correction (Wick et al., 2019). Therefore, it is necessary to polish with short reads to obtain high-quality genome sequences (De Maio et al., 2019). Here, we evaluated whether a genome sequence assembled using only ONT long reads could be used to identify bacterial species with KITSUNE.

**FIGURE 3:**
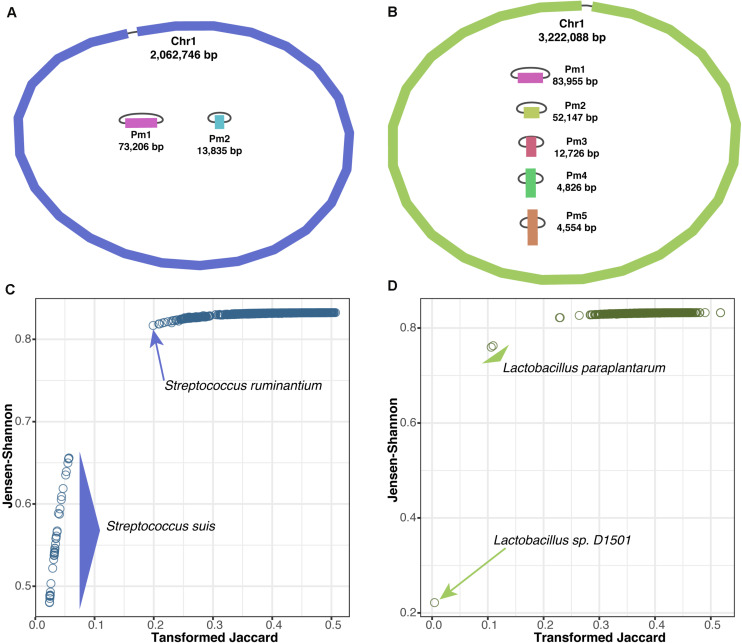
Bacterial species identification based on genome assembly via long-read sequencing. **(A)** Illustration of assembled chromosome and plasmids of *S. suis* HU_SS30 and **(B)** *L. plantarum* BCC9546. Scatter plots of genomic distances derived from of genomes pairs (Jensen–Shannon vs. transformed Jaccard methods) for *S. suis* **(C)** and *L. plantarum* **(D)** queried against the 8,861 complete bacterial genomes.

We performed whole-genome sequencing and *de novo* assembly for two bacterial species, *S. suis* HU_SS30 and *L. plantarum* BCC9546, using only ONT long-read sequencing. We generated sequencing depths of ∼53 × (146 Mb) for *S. suis* and ∼45 × (177 Mb) for *L. plantarum*. The assembled genomes are illustrated in Figures 3A,B. For *S. suis*, the *de novo* assembly yielded three circular contigs of a chromosome of approximately 2 Mb and two plasmids of approximately 73 and 14 kb. For *L. plantarum*, the *de novo* assembly yielded six circular contigs of a chromosome of approximately 3.2 Mb and five plasmids ranging in size from 4.5 to 84 kb. Only the assembled chromosome sequences were used for species identification by querying the sequences against our dataset of 8,861 complete bacterial genomes.

We used KITSUNE to calculate the genomic distances at our identified empirically optimal k-mer length of 17 (Figure 1) using the Jensen–Shannon method and the Jaccard method with transformation (Fan et al., 2015), which previously showed a non-linear relationship between them (Figure 2B), and compared them as illustrated in Figures 3C,D for *S. suis* and *L. plantarum*, respectively. By considering the nearest-neighbor genomes within the complete bacterial genome dataset, we could identify *S. suis* based on the clear cluster of genomic distances within the species (<0.06 for transformed Jaccard and <0.66 for Jensen–Shannon). For *L. plantarum*, we identified the unclassified *Lactobacillus* sp. *D1501* as the closest species, as the complete bacterial genome dataset used here lacked *L. plantarum*, indicating the importance of a database for species identification.

### Evaluation of Different K-mer Lengths for Fungal Species Identification

**FIGURE 4:**
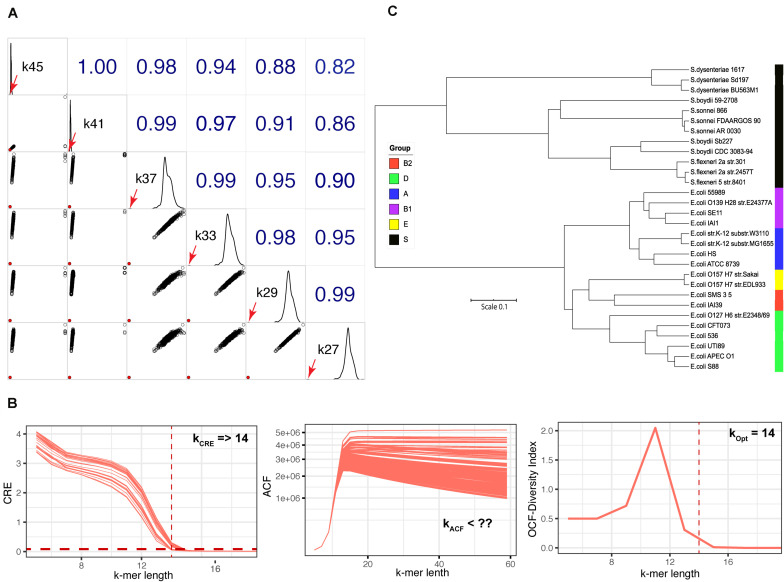
Fungal species identification and k-mer length identification of the *E. coli-Shigella* dataset. **(A)** Correlogram plot for identifying the fungal species *S. cerevisiae* CEN.PK 113-7D by querying against the dataset of 729 fungal genomes at k-mer lengths of 27–45 using the transformed Jaccard genomic distance. Lower left triangle: scatter plots of the genomic distance for different k-mer lengths; solid red circle is the shortest genomic distance between *S. cerevisiae* CEN.PK 113-7D and the species found in the fungal genome dataset (*S. cerevisiae* S288C). Upper right triangle: Spearman’s rank correlation coefficients among different k-mer lengths; diagonal boxes show the distribution of genomic distances. The diagonal boxes show the distribution of the genomics distances; the red arrows represent the genomic distance between *S. cerevisiae* strain CEN.PK 113-7D and S288C. **(B)** The plots of CRE, ACF, and OFC for the 30 strains of *E. coli–Shigella* dataset used to identify empirically optimal k-mer length. **(C)** Tree constructed from genomic distances based on the d2Star method using Afann at the identified empirically optimal k-mer length of 14. Phylogroups are indicated by colored boxes on the right side of the tree: black, *Shigella* spp.; blue, A; purple, B1; red, B2; green, D; and yellow, E.

Unlike with the viral or bacterial genome datasets, we did not obtain a unique empirically optimal k-mer length from the fungal genome dataset (Figure 1). Therefore, we explored the impact of different k-mer lengths on identifying fungal species, using *S. cerevisiae* strain CEN.PK113-7D as an example. We used the transformed Jaccard index (see Eq. 5) to calculate the genomic distance for *S. cerevisiae* using k-mer lengths of between 27–45 (the range of empirically optimal k-mer lengths defined in Figure 1E) against the genomes in the dataset of 729 fungal genomes (Figure 4A). With this approach, we were able to identify the species *S. cerevisiae* at any k-mer length based on the shortest distance. We observed that the genomic distance, calculated at k-mer lengths ≥41, between *S. cerevisiae* strain CEN.PK113-7D and strain S288C was closer to the other distances derived *S. cerevisiae* CEN.PK113-7D and other fungi genomes in the reference dataset when compared with at k-mer lengths <37 (diagonal plots of Figure 4A). This indicated that the discrimination power decreased at k-mer lengths ≥41.

### Using KITSUNE to Identify Empirically Optimal K-mer Length for Closely Related Bacterial Species

The three-step approach to identify empirically optimal k-mer length was first designed for inter species comparison. We evaluated whether we could use KITSUNE to identify empirically optimal k-mer length for the closely related species of *E. coli- Shigella*, which is a classic problem in microbiology for species differentiation. We calculated CRE, ACF, and OCF for the 30 genomes in the *E. coli-Shigella* dataset as illustrated in Figure 4B. The minimum k-mer length of 14 was identified based on CRE. The ACF, which is used to evaluate the level of common features among the considered genomes, decreased slowly as k-mer length increased because of the high similarity of the genome sequences. Therefore, the maximum k-mer length could not be identified with ACF. However, the OCF indicated that a k-mer length of 14 was the empirically optimal k-mer length because it gave the highest diversity index (*H*). We then calculated the genomic distances based on d2Star method using Afann software and used them to constructed a tree as illustrated in Figure 4C. We observed a clear separation among groups of *E. coli* and *Shigella* spp. Moreover, different phylo-types of *E. coli* were well discriminated at the identified empirically optimal k-mer length.

### Virus Species-Specific Sequence Identification Using ACF

Taxon-specific sequences have been used for rapid species classification, taxonomic rank identification, functional inference, and taxon abundance estimation for genome and metagenome samples (Truong et al., 2015; Wood et al., 2019). With this in mind, we demonstrated the capability of KITSUNE to identify the shortest k-mer length that is unique for individual viral species based on the genome sequence by applying the ACF, which can be used to identify common features of any two genomes. We selected RNA/DNA viruses associated with infectious disease epidemics/pandemics as described in the Methods section. Using the ACF for k-mer lengths of 7–51, the individual viral genomes were queried against the reference viral genome dataset (8,967 genomes) to evaluate whether the genomes had common sequences.

**FIGURE 5:**
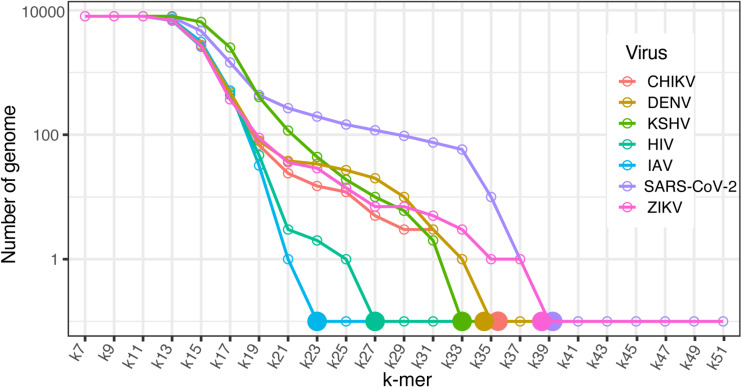
Virus specific sequence identification. Line plot of the number of genomes within the dataset of 8,967 viral reference genomes that have sequences in common with the seven selected viruses across k-mer lengths of 57–51. Solid dots represent the shortest unique k-mer of individual viruses. CHIKV, Chikungunya virus; DENV, Dengue virus; KSHV, Kaposi’s sarcoma-associated human herpes virus; HIV, human immunodeficiency virus; IAV, influenza A virus; SARS-CoV-2, severe acute respiratory syndrome coronavirus 2; and ZIKV, Zika virus.

The frequency of genomes with sequences in common with the seven selected viruses was recorded for each k-mer length and plotted for comparison (Figure 5). Based on this graph, the shortest k-mer length that was unique for the selected viruses was identified; these lengths were 23 for IAV, 27 for HIV, 33 for KHSV, and 35 for DENV, CHIKV, and 39 for SARS-CoV-2, ZIKV. We assessed the specificity of these shortest unique k-mers with a BLAST search against the NCBI nr/nt database, which includes sequences from various organisms. The BLAST results based on the top 50 hits of randomly selected sequences for the viruses of interest showed very good specificity for the shortest unique k-mers for species identification (see Supplementary Material).

## Discussion

Although k-mer length is critical, few studies have endeavored to determine empirically optimal k-mer lengths for making alignment-free genomic comparisons. KITSUNE provides a systematic way to investigate empirically optimal k-mer lengths, and it can be used for many applications. Here, we applied KITSUNE to identify the empirically optimal k-mer length for large-scale datasets of viral, bacterial, and fungal genomes through random sampling. The subsample size, which is an important parameter, needs to be large enough to represent the population of considered genomes. This approach gave consistent results for empirically optimal k-mer length for the viral and bacterial datasets, but not for the fungal dataset, which contained larger and more diverse genomes, indicating an insufficient subsample size. The k-mer–based information content within a genome is highly dependent on genome size (Zhang et al., 2017); therefore, long k-mers are necessary to identify the empirically optimal k-mer length. Long k-mers length produces a large, highly complex search space, which increases exponentially by a factor of 4^*k*^ for nucleotide sequence.

We found that the genomic distance calculation was quite consistent across methods in terms of ranking (Figure 2B) and could be used for species identification (Figures 3C,D, 4A). KITSUNE requires more computational time to calculate genomic distances than the Mash method (Ondov et al., 2016) because KITSUNE uses uncompressed k-mer frequency data. However, the MinHash/Sketching algorithm (Broder, 1977; Indyk and Motwani, 1998) uses lossy compression to represent k-mers, which allows only the Jaccard index to be used to determine genomic distance. Moreover, sequence information cannot be retrieved after it is compressed by MinHash, so it is not possible to identify actual nucleotide sequences within a k-mer frequency profile. Such actual nucleotide sequences are very useful for identifying genetic sequence markers, as demonstrated in Figure 5 for viruses using KITSUNE. These are the trade-offs between computational time and the resolution of sequence data.

The specific characteristic of genomic distance profile derived from individual method was observed especially by Jensen–Shannon, d2Star and Cosine (Figure 2B). This raises an important research question about how to calibrate genomic distances derived from alignment-free methods with standard phylogenetic analysis, which has a robust statistical model for in-depth evolutionary analysis, and use the genomic distances derived from alignment-free analysis to study evolution. Nevertheless, the alignment-free genomic distance yielded rapid and accurate species identification.

Researchers can use KITSUNE to systematically identify the empirically optimal k-mer length for genomes of interest based on the three-step approach. Calculating ACF, which must be done for all possible pairs of genomes, and OFC, which is an all-genomes comparison, is computationally intensive. A subsampling approach reduces this computational load and gives a good approximation of empirically optimal k-mer length. Even though KITSUNE was developed for genomes of different species, it can be used to analyze closely related species by ignoring the ACF. KITSUNE uses the assembled genomes, not sequencing reads, to identify the empirically optimal k-mer length. Nevertheless, the identified empirically optimal k-mer length in Figure 1 for the three kingdoms and taxa-specific k-mer as demonstrated in Figure 5. KITSUNE can be used to calculate genomic distances that can be used for many applications. However, we implemented the genomic distance methods based on available distance functions in the Python environment. Genomic distance is important for comparative genomics using an alignment-free approach; therefore, we recommended the users explore advanced genomic distance calculations from the published literature.

## Conclusion

In summary, we present KITSUNE, an open source software that can be used to identify the empirically optimal k-mer length for phylogenomic analysis of a given set of genomes and for estimating genomic distances and identifying taxon-specific sequences. Thus, KITSUNE is an alternative alignment-free tool for comparative genomics.

## Author’s Note

This paper is dedicated to the memory of IN’s beloved dog RAME, who inspired the KITSUNE logo.

## Data Availability Statement

The datasets presented in this study can be found in online repositories. Sequence Read Archive (SRA) database under BioProject number PRJNA644942. Accession number(s) can be found below: https://www.ncbi.nlm.nih.gov/, PRJNA398797.

## Author Contributions

IN conceived and directed the project and wrote the manuscript. NP developed and implemented the software. DA participated in the software development and implementation. PP developed and implemented the software in the beginning stage. IN, DA, and PJ performed the data analysis. TW, SY, and NC performed the Oxford Nanopore Technologies sequencing. SY and NC performed the bacterial culture and DNA extraction. S-RJ provided technical assistance with the methodology. All authors read and approved the final manuscript.

## Conflict of Interest

The authors declare that the research was conducted in the absence of any commercial or financial relationships that could be construed as a potential conflict of interest.

## Abbreviations

ACF: average number of common features
CHIKV: Chikungunya virus
CRE: cumulative relative entropy
*H*: Shannon diversity index
HIV: human immunodeficiency virus 1
IAV: influenza A virus
KITSUNE: K-mer –length Iterative Selection for UNbiased Ecophylogenomics
KSHV: Kaposi’s sarcoma-associated human herpes virus
MRS: Man Rogosa and Sharpe
NCBI: National Center for Biotechnology Information
OCF: observed common features
SARS-CoV-2: severe acute respiratory syndrome coronavirus 2
ZIKV: Zika virus.
