# Supplementary material for: KITSUNE: A Tool for Identifying Empirically Optimal K-mer Length for Alignment-Free Phylogenomic Analysis
Source: Front Bioeng Biotechnol. 2020 Sep 23;8:556413. doi: 10.3389/fbioe.2020.556413 (PMC7538862; doi:10.3389/fbioe.2020.556413)
Supplement: Supplementary file 1 [file Data_Sheet_1.PDF]

Top 50 highest score hit NCBI blast result of randomly select two sequences of the unique shortest k-mer length identified for the individual selected seven virus as shown in Figure 4B.

SAR-COV-2 K39

AGAATCATACATCACCAGATGTTGATTTAGGTGACATCT

Descriptions | Graphic Summary | Alignments | **Taxonomy**

Reports | **Lineage** | **Organism** | **Taxonomy**

100 sequences selected

Organism	Blast Name	Score	Number of Hits	Description
Severe acute respiratory syndrome coronavirus 2	viruses	73.1	102	Severe acute respiratory syndrome coronavirus 2 h

	Description	Max Score	Total Score	Query Cover	E value	Per. Ident	Accession
☑	Severe acute respiratory syndrome coronavirus 2 isolate SARS-CoV-2/human/USA/VA-DCL!	73.1	73.1	100%	2e-10	100.00%	MT322424.1
☑	Severe acute respiratory syndrome coronavirus 2 isolate SARS-CoV-2/human/USA/VA-DCL!	73.1	73.1	100%	2e-10	100.00%	MT322423.1
☑	Severe acute respiratory syndrome coronavirus 2 isolate SARS-CoV-2/human/USA/VA-DCL!	73.1	73.1	100%	2e-10	100.00%	MT322422.1
☑	Severe acute respiratory syndrome coronavirus 2 isolate SARS-CoV-2/human/USA/VA-DCL!	73.1	73.1	100%	2e-10	100.00%	MT322421.1
☑	Severe acute respiratory syndrome coronavirus 2 isolate SARS-CoV-2/human/USA/VA-DCL!	73.1	73.1	100%	2e-10	100.00%	MT322420.1
☑	Severe acute respiratory syndrome coronavirus 2 isolate SARS-CoV-2/human/USA/VA-DCL!	73.1	73.1	100%	2e-10	100.00%	MT322419.1
☑	Severe acute respiratory syndrome coronavirus 2 isolate SARS-CoV-2/human/USA/VA-DCL!	73.1	73.1	100%	2e-10	100.00%	MT322418.1
☑	Severe acute respiratory syndrome coronavirus 2 isolate SARS-CoV-2/human/USA/VA-DCL!	73.1	73.1	100%	2e-10	100.00%	MT322417.1
☑	Severe acute respiratory syndrome coronavirus 2 isolate SARS-CoV-2/human/USA/VA-DCL!	73.1	73.1	100%	2e-10	100.00%	MT322416.1
☑	Severe acute respiratory syndrome coronavirus 2 isolate SARS-CoV-2/human/USA/VA-DCL!	73.1	73.1	100%	2e-10	100.00%	MT322415.1
☑	Severe acute respiratory syndrome coronavirus 2 isolate SARS-CoV-2/human/USA/VA-DCL!	73.1	73.1	100%	2e-10	100.00%	MT322414.1
☑	Severe acute respiratory syndrome coronavirus 2 isolate SARS-CoV-2/human/USA/VA-DCL!	73.1	73.1	100%	2e-10	100.00%	MT322413.1
☑	Severe acute respiratory syndrome coronavirus 2 isolate SARS-CoV-2/human/USA/VA-DCL!	73.1	73.1	100%	2e-10	100.00%	MT322412.1
☑	Severe acute respiratory syndrome coronavirus 2 isolate SARS-CoV-2/human/USA/VA-DCL!	73.1	73.1	100%	2e-10	100.00%	MT322411.1
☑	Severe acute respiratory syndrome coronavirus 2 isolate SARS-CoV-2/human/USA/VA-DCL!	73.1	73.1	100%	2e-10	100.00%	MT322410.1
☑	Severe acute respiratory syndrome coronavirus 2 isolate SARS-CoV-2/human/USA/VA-DCL!	73.1	73.1	100%	2e-10	100.00%	MT322409.1
☑	Severe acute respiratory syndrome coronavirus 2 isolate SARS-CoV-2/human/USA/VA-DCL!	73.1	73.1	100%	2e-10	100.00%	MT322408.1
☑	Severe acute respiratory syndrome coronavirus 2 isolate SARS-CoV-2/human/USA/VA-DCL!	73.1	73.1	100%	2e-10	100.00%	MT322407.1
☑	Severe acute respiratory syndrome coronavirus 2 isolate SARS-CoV-2/human/USA/VA-DCL!	73.1	73.1	100%	2e-10	100.00%	MT322406.1
☑	Severe acute respiratory syndrome coronavirus 2 isolate SARS-CoV-2/human/USA/VA-DCL!	73.1	73.1	100%	2e-10	100.00%	MT322405.1
☑	Severe acute respiratory syndrome coronavirus 2 isolate SARS-CoV-2/human/USA/VA-DCL!	73.1	73.1	100%	2e-10	100.00%	MT322404.1
☑	Severe acute respiratory syndrome coronavirus 2 isolate SARS-CoV-2/human/USA/VA-DCL!	73.1	73.1	100%	2e-10	100.00%	MT322403.1
☑	Severe acute respiratory syndrome coronavirus 2 isolate SARS-CoV-2/human/USA/VA-DCL!	73.1	73.1	100%	2e-10	100.00%	MT322402.1
☑	Severe acute respiratory syndrome coronavirus 2 isolate SARS-CoV-2/human/USA/VA-DCL!	73.1	73.1	100%	2e-10	100.00%	MT322401.1
☑	Severe acute respiratory syndrome coronavirus 2 isolate SARS-CoV-2/human/USA/VA-DCL!	73.1	73.1	100%	2e-10	100.00%	MT322400.1
☑	Severe acute respiratory syndrome coronavirus 2 isolate SARS-CoV-2/human/USA/VA-DCL!	73.1	73.1	100%	2e-10	100.00%	MT322399.1
☑	Severe acute respiratory syndrome coronavirus 2 isolate SARS-CoV-2/human/USA/VA-DCL!	73.1	73.1	100%	2e-10	100.00%	MT322398.1
☑	Severe acute respiratory syndrome coronavirus 2 isolate SARS-CoV-2/human/USA/VA-DCL!	73.1	73.1	100%	2e-10	100.00%	MT322397.1
☑	Severe acute respiratory syndrome coronavirus 2 isolate SARS-CoV-2/human/USA/VA-DCL!	73.1	73.1	100%	2e-10	100.00%	MT322396.1
☑	Severe acute respiratory syndrome coronavirus 2 isolate SARS-CoV-2/human/USA/VA-DCL!	73.1	73.1	100%	2e-10	100.00%	MT322395.1
☑	Severe acute respiratory syndrome coronavirus 2 isolate SARS-CoV-2/human/USA/VA-DCL!	73.1	73.1	100%	2e-10	100.00%	MT322394.1
☑	Severe acute respiratory syndrome coronavirus 2 isolate SARS-CoV-2/human/ISR/ISR_IT03	73.1	73.1	100%	2e-10	100.00%	MT276598.1
☑	Severe acute respiratory syndrome coronavirus 2 isolate SARS-CoV-2/human/ISR/ISR_JP0:	73.1	73.1	100%	2e-10	100.00%	MT276597.1
☑	Severe acute respiratory syndrome coronavirus 2 isolate SARS-CoV-2/human/USA/TX_202(	73.1	73.1	100%	2e-10	100.00%	MT276331.1
☑	Severe acute respiratory syndrome coronavirus 2 isolate SARS-CoV-2/human/USA/FL_5125	73.1	73.1	100%	2e-10	100.00%	MT276329.1
☑	Severe acute respiratory syndrome coronavirus 2 isolate SARS-CoV-2/human/USA/OR_265(	73.1	73.1	100%	2e-10	100.00%	MT276328.1
☑	Severe acute respiratory syndrome coronavirus 2 isolate SARS-CoV-2/human/USA/GA_274:	73.1	73.1	100%	2e-10	100.00%	MT276327.1
☑	Severe acute respiratory syndrome coronavirus 2 isolate SARS-CoV-2/human/USA/GA_274'	73.1	73.1	100%	2e-10	100.00%	MT276326.1
☑	Severe acute respiratory syndrome coronavirus 2 isolate SARS-CoV-2/human/USA/CA_260:	73.1	73.1	100%	2e-10	100.00%	MT276324.1
☑	Severe acute respiratory syndrome coronavirus 2 isolate SARS-CoV-2/human/USA/RI_0520	73.1	73.1	100%	2e-10	100.00%	MT276323.1
☑	Severe acute respiratory syndrome coronavirus 2 isolate SARS-CoV-2/human/USA/WA-UW:	73.1	73.1	100%	2e-10	100.00%	MT263469.1
☑	Severe acute respiratory syndrome coronavirus 2 isolate SARS-CoV-2/human/USA/WA-UW:	73.1	73.1	100%	2e-10	100.00%	MT263468.1
☑	Severe acute respiratory syndrome coronavirus 2 isolate SARS-CoV-2/human/USA/WA-UW:	73.1	73.1	100%	2e-10	100.00%	MT263467.1
☑	Severe acute respiratory syndrome coronavirus 2 isolate SARS-CoV-2/human/USA/WA-UW:	73.1	73.1	100%	2e-10	100.00%	MT263466.1
☑	Severe acute respiratory syndrome coronavirus 2 isolate SARS-CoV-2/human/USA/WA-UW:	73.1	73.1	100%	2e-10	100.00%	MT263465.1
☑	Severe acute respiratory syndrome coronavirus 2 isolate SARS-CoV-2/human/USA/WA-UW:	73.1	73.1	100%	2e-10	100.00%	MT263464.1
☑	Severe acute respiratory syndrome coronavirus 2 isolate SARS-CoV-2/human/USA/WA-UW:	73.1	73.1	100%	2e-10	100.00%	MT263463.1
☑	Severe acute respiratory syndrome coronavirus 2 isolate SARS-CoV-2/human/USA/WA-UW:	73.1	73.1	100%	2e-10	100.00%	MT263462.1
☑	Severe acute respiratory syndrome coronavirus 2 isolate SARS-CoV-2/human/USA/WA-UW:	73.1	73.1	100%	2e-10	100.00%	MT263461.1
☑	Severe acute respiratory syndrome coronavirus 2 isolate SARS-CoV-2/human/USA/WA-UW:	73.1	73.1	100%	2e-10	100.00%	MT263460.1

SAR-COV-2 K39

GTTACTTTCCAAAGTGCAGTGAAAAGAACAATCAAGGGT

Descriptions | Graphic Summary | Alignments | **Taxonomy**

Reports | **Lineage** | Organism | Taxonomy

50 sequences selected

Organism	Blast Name	Score	Number of Hits	Description
Severe acute respiratory syndrome coronavirus 2	viruses	73.1	52	Severe acute respiratory syndrome coronavirus 2 h

	Description	Max Score	Total Score	Query Cover	E value	Per. Ident	Accession
☑	Severe acute respiratory syndrome coronavirus 2 isolate SARS-CoV-2/human/USA/VA-DCL	73.1	73.1	100%	2e-10	100.00%	MT322424.1
☑	Severe acute respiratory syndrome coronavirus 2 isolate SARS-CoV-2/human/USA/VA-DCL	73.1	73.1	100%	2e-10	100.00%	MT322423.1
☑	Severe acute respiratory syndrome coronavirus 2 isolate SARS-CoV-2/human/USA/VA-DCL	73.1	73.1	100%	2e-10	100.00%	MT322422.1
☑	Severe acute respiratory syndrome coronavirus 2 isolate SARS-CoV-2/human/USA/VA-DCL	73.1	73.1	100%	2e-10	100.00%	MT322421.1
☑	Severe acute respiratory syndrome coronavirus 2 isolate SARS-CoV-2/human/USA/VA-DCL	73.1	73.1	100%	2e-10	100.00%	MT322420.1
☑	Severe acute respiratory syndrome coronavirus 2 isolate SARS-CoV-2/human/USA/VA-DCL	73.1	73.1	100%	2e-10	100.00%	MT322419.1
☑	Severe acute respiratory syndrome coronavirus 2 isolate SARS-CoV-2/human/USA/VA-DCL	73.1	73.1	100%	2e-10	100.00%	MT322418.1
☑	Severe acute respiratory syndrome coronavirus 2 isolate SARS-CoV-2/human/USA/VA-DCL	73.1	73.1	100%	2e-10	100.00%	MT322417.1
☑	Severe acute respiratory syndrome coronavirus 2 isolate SARS-CoV-2/human/USA/VA-DCL	73.1	73.1	100%	2e-10	100.00%	MT322416.1
☑	Severe acute respiratory syndrome coronavirus 2 isolate SARS-CoV-2/human/USA/VA-DCL	73.1	73.1	100%	2e-10	100.00%	MT322415.1
☑	Severe acute respiratory syndrome coronavirus 2 isolate SARS-CoV-2/human/USA/VA-DCL	73.1	73.1	100%	2e-10	100.00%	MT322414.1
☑	Severe acute respiratory syndrome coronavirus 2 isolate SARS-CoV-2/human/USA/VA-DCL	73.1	73.1	100%	2e-10	100.00%	MT322413.1
☑	Severe acute respiratory syndrome coronavirus 2 isolate SARS-CoV-2/human/USA/VA-DCL	73.1	73.1	100%	2e-10	100.00%	MT322412.1
☑	Severe acute respiratory syndrome coronavirus 2 isolate SARS-CoV-2/human/USA/VA-DCL	73.1	73.1	100%	2e-10	100.00%	MT322411.1
☑	Severe acute respiratory syndrome coronavirus 2 isolate SARS-CoV-2/human/USA/VA-DCL	73.1	73.1	100%	2e-10	100.00%	MT322410.1
☑	Severe acute respiratory syndrome coronavirus 2 isolate SARS-CoV-2/human/USA/VA-DCL	73.1	73.1	100%	2e-10	100.00%	MT322409.1
☑	Severe acute respiratory syndrome coronavirus 2 isolate SARS-CoV-2/human/USA/VA-DCL	73.1	73.1	100%	2e-10	100.00%	MT322408.1
☑	Severe acute respiratory syndrome coronavirus 2 isolate SARS-CoV-2/human/USA/VA-DCL	73.1	73.1	100%	2e-10	100.00%	MT322407.1
☑	Severe acute respiratory syndrome coronavirus 2 isolate SARS-CoV-2/human/USA/VA-DCL	73.1	73.1	100%	2e-10	100.00%	MT322406.1
☑	Severe acute respiratory syndrome coronavirus 2 isolate SARS-CoV-2/human/USA/VA-DCL	73.1	73.1	100%	2e-10	100.00%	MT322405.1
☑	Severe acute respiratory syndrome coronavirus 2 isolate SARS-CoV-2/human/USA/VA-DCL	73.1	73.1	100%	2e-10	100.00%	MT322404.1
☑	Severe acute respiratory syndrome coronavirus 2 isolate SARS-CoV-2/human/USA/VA-DCL	73.1	73.1	100%	2e-10	100.00%	MT322403.1
☑	Severe acute respiratory syndrome coronavirus 2 isolate SARS-CoV-2/human/USA/VA-DCL	73.1	73.1	100%	2e-10	100.00%	MT322402.1
☑	Severe acute respiratory syndrome coronavirus 2 isolate SARS-CoV-2/human/USA/VA-DCL	73.1	73.1	100%	2e-10	100.00%	MT322401.1
☑	Severe acute respiratory syndrome coronavirus 2 isolate SARS-CoV-2/human/USA/VA-DCL	73.1	73.1	100%	2e-10	100.00%	MT322400.1
☑	Severe acute respiratory syndrome coronavirus 2 isolate SARS-CoV-2/human/USA/VA-DCL	73.1	73.1	100%	2e-10	100.00%	MT322399.1
☑	Severe acute respiratory syndrome coronavirus 2 isolate SARS-CoV-2/human/USA/VA-DCL	73.1	73.1	100%	2e-10	100.00%	MT322398.1
☑	Severe acute respiratory syndrome coronavirus 2 isolate SARS-CoV-2/human/USA/VA-DCL	73.1	73.1	100%	2e-10	100.00%	MT322397.1
☑	Severe acute respiratory syndrome coronavirus 2 isolate SARS-CoV-2/human/USA/VA-DCL	73.1	73.1	100%	2e-10	100.00%	MT322396.1
☑	Severe acute respiratory syndrome coronavirus 2 isolate SARS-CoV-2/human/USA/VA-DCL	73.1	73.1	100%	2e-10	100.00%	MT322395.1
☑	Severe acute respiratory syndrome coronavirus 2 isolate SARS-CoV-2/human/USA/VA-DCL	73.1	73.1	100%	2e-10	100.00%	MT322394.1
☑	Severe acute respiratory syndrome coronavirus 2 isolate SARS-CoV-2/human/ISR/ISR_IT03	73.1	73.1	100%	2e-10	100.00%	MT276598.1
☑	Severe acute respiratory syndrome coronavirus 2 isolate SARS-CoV-2/human/ISR/ISR_JP0	73.1	73.1	100%	2e-10	100.00%	MT276597.1
☑	Severe acute respiratory syndrome coronavirus 2 isolate SARS-CoV-2/human/USA/TX_202	73.1	73.1	100%	2e-10	100.00%	MT276331.1
☑	Severe acute respiratory syndrome coronavirus 2 isolate SARS-CoV-2/human/USA/FL_5125	73.1	73.1	100%	2e-10	100.00%	MT276329.1
☑	Severe acute respiratory syndrome coronavirus 2 isolate SARS-CoV-2/human/USA/OR_265	73.1	73.1	100%	2e-10	100.00%	MT276328.1
☑	Severe acute respiratory syndrome coronavirus 2 isolate SARS-CoV-2/human/USA/GA_274	73.1	73.1	100%	2e-10	100.00%	MT276327.1
☑	Severe acute respiratory syndrome coronavirus 2 isolate SARS-CoV-2/human/USA/GA_274	73.1	73.1	100%	2e-10	100.00%	MT276326.1
☑	Severe acute respiratory syndrome coronavirus 2 isolate SARS-CoV-2/human/USA/CA_260	73.1	73.1	100%	2e-10	100.00%	MT276324.1
☑	Severe acute respiratory syndrome coronavirus 2 isolate SARS-CoV-2/human/USA/RI_0520	73.1	73.1	100%	2e-10	100.00%	MT276323.1
☑	Severe acute respiratory syndrome coronavirus 2 isolate SARS-CoV-2/human/USA/WA-UW	73.1	73.1	100%	2e-10	100.00%	MT263469.1
☑	Severe acute respiratory syndrome coronavirus 2 isolate SARS-CoV-2/human/USA/WA-UW	73.1	73.1	100%	2e-10	100.00%	MT263468.1
☑	Severe acute respiratory syndrome coronavirus 2 isolate SARS-CoV-2/human/USA/WA-UW	73.1	73.1	100%	2e-10	100.00%	MT263467.1
☑	Severe acute respiratory syndrome coronavirus 2 isolate SARS-CoV-2/human/USA/WA-UW	73.1	73.1	100%	2e-10	100.00%	MT263466.1
☑	Severe acute respiratory syndrome coronavirus 2 isolate SARS-CoV-2/human/USA/WA-UW	73.1	73.1	100%	2e-10	100.00%	MT263465.1
☑	Severe acute respiratory syndrome coronavirus 2 isolate SARS-CoV-2/human/USA/WA-UW	73.1	73.1	100%	2e-10	100.00%	MT263464.1
☑	Severe acute respiratory syndrome coronavirus 2 isolate SARS-CoV-2/human/USA/WA-UW	73.1	73.1	100%	2e-10	100.00%	MT263463.1
☑	Severe acute respiratory syndrome coronavirus 2 isolate SARS-CoV-2/human/USA/WA-UW	73.1	73.1	100%	2e-10	100.00%	MT263462.1
☑	Severe acute respiratory syndrome coronavirus 2 isolate SARS-CoV-2/human/USA/WA-UW	73.1	73.1	100%	2e-10	100.00%	MT263461.1
☑	Severe acute respiratory syndrome coronavirus 2 isolate SARS-CoV-2/human/USA/WA-UW	73.1	73.1	100%	2e-10	100.00%	MT263460.1

Zika K39

TGAAGCCATAAAAACAAGACTCCGTACTGTGATCTTAGC

Descriptions | Graphic Summary | Alignments | **Taxonomy**

Reports **Lineage** **Organism** **Taxonomy**

100 sequences selected

Organism	Blast Name	Score	Number of Hits	Description
Zika virus	viruses	73.1	101	Zika virus hits

Description	Max Score	Total Score	Query Cover	E value	Per. Ident	Accession
Zika virus isolate JXLHS1807 polyprotein gene, complete cds	73.1	73.1	100%	2e-10	100.00%	MK696551.1
Zika virus isolate 15098, complete genome	73.1	73.1	100%	2e-10	100.00%	MK566202.1
Zika virus PE243 polyprotein (POLY) gene, partial cds	73.1	73.1	100%	2e-10	100.00%	MT078742.1
Zika virus PE243 PE-innoculum polyprotein (POLY) gene, partial cds	73.1	73.1	100%	2e-10	100.00%	MT078741.1
Zika virus PE243 mouse-adapted 1 polyprotein (POLY) gene, partial cds	73.1	73.1	100%	2e-10	100.00%	MT078739.1
Zika virus isolate FLUR100 polyprotein gene, complete cds	73.1	73.1	100%	2e-10	100.00%	MK269359.1
Zika virus isolate FLSR077 polyprotein gene, complete cds	73.1	73.1	100%	2e-10	100.00%	MK269354.1
Zika virus isolate Z02 polyprotein gene, partial cds	73.1	73.1	100%	2e-10	100.00%	MK216748.1
Zika virus isolate Z03 polyprotein gene, partial cds	73.1	73.1	100%	2e-10	100.00%	MK216747.1
Zika virus isolate AMA302 polyprotein gene, complete cds	73.1	73.1	100%	2e-10	100.00%	MK216745.1
Zika virus isolate AMA91 polyprotein gene, partial cds	73.1	73.1	100%	2e-10	100.00%	MK216744.1
Zika virus isolate AMA88 polyprotein gene, complete cds	73.1	73.1	100%	2e-10	100.00%	MK216743.1
Zika virus isolate AMA90 polyprotein gene, complete cds	73.1	73.1	100%	2e-10	100.00%	MK216742.1
Zika virus isolate AMA89 polyprotein gene, complete cds	73.1	73.1	100%	2e-10	100.00%	MK216741.1
Zika virus isolate AMA85 polyprotein gene, partial cds	73.1	73.1	100%	2e-10	100.00%	MK216740.1
Zika virus isolate AMA82 polyprotein gene, partial cds	73.1	73.1	100%	2e-10	100.00%	MK216738.1
Zika virus isolate AMA84 polyprotein gene, partial cds	73.1	73.1	100%	2e-10	100.00%	MK216736.1
Zika virus isolate AMA71 polyprotein gene, partial cds	73.1	73.1	100%	2e-10	100.00%	MK216734.1
Zika virus isolate AMA70 polyprotein gene, partial cds	73.1	73.1	100%	2e-10	100.00%	MK216731.1
Zika virus isolate AMA67 polyprotein gene, partial cds	73.1	73.1	100%	2e-10	100.00%	MK216730.1
Zika virus isolate AMA66 polyprotein gene, partial cds	73.1	73.1	100%	2e-10	100.00%	MK216728.1
Zika virus isolate AMA65 polyprotein gene, complete cds	73.1	73.1	100%	2e-10	100.00%	MK216727.1
Zika virus isolate AMA62 polyprotein gene, partial cds	73.1	73.1	100%	2e-10	100.00%	MK216726.1
Zika virus isolate AMA55 polyprotein gene, partial cds	73.1	73.1	100%	2e-10	100.00%	MK216725.1
Zika virus isolate AMA57 polyprotein gene, partial cds	73.1	73.1	100%	2e-10	100.00%	MK216723.1
Zika virus isolate AMA58 polyprotein gene, partial cds	73.1	73.1	100%	2e-10	100.00%	MK216722.1
Zika virus isolate AMA53 polyprotein gene, partial cds	73.1	73.1	100%	2e-10	100.00%	MK216721.1
Zika virus isolate AMA51 polyprotein gene, partial cds	73.1	73.1	100%	2e-10	100.00%	MK216720.1
Zika virus isolate AMA50 polyprotein gene, partial cds	73.1	73.1	100%	2e-10	100.00%	MK216719.1
Zika virus isolate AMA49 polyprotein gene, partial cds	73.1	73.1	100%	2e-10	100.00%	MK216718.1
Zika virus isolate AMA48 polyprotein gene, partial cds	73.1	73.1	100%	2e-10	100.00%	MK216717.1
Zika virus isolate AMA47 polyprotein gene, partial cds	73.1	73.1	100%	2e-10	100.00%	MK216716.1
Zika virus isolate AMA46 polyprotein gene, partial cds	73.1	73.1	100%	2e-10	100.00%	MK216715.1
Zika virus isolate AMA44 polyprotein gene, partial cds	73.1	73.1	100%	2e-10	100.00%	MK216714.1
Zika virus isolate AMA45 polyprotein gene, complete cds	73.1	73.1	100%	2e-10	100.00%	MK216713.1
Zika virus isolate AMA42 polyprotein gene, partial cds	73.1	73.1	100%	2e-10	100.00%	MK216712.1
Zika virus isolate AMA41 polyprotein gene, complete cds	73.1	73.1	100%	2e-10	100.00%	MK216710.1
Zika virus isolate AMA37 polyprotein gene, partial cds	73.1	73.1	100%	2e-10	100.00%	MK216709.1
Zika virus isolate AMA27 polyprotein gene, partial cds	73.1	73.1	100%	2e-10	100.00%	MK216703.1
Zika virus isolate AMA22 polyprotein gene, complete cds	73.1	73.1	100%	2e-10	100.00%	MK216699.1
Zika virus isolate AMA21 polyprotein gene, complete cds	73.1	73.1	100%	2e-10	100.00%	MK216698.1
Zika virus isolate AMA20 polyprotein gene, complete cds	73.1	73.1	100%	2e-10	100.00%	MK216697.1
Zika virus isolate AMA19 polyprotein gene, complete cds	73.1	73.1	100%	2e-10	100.00%	MK216696.1
Zika virus isolate AMA16 polyprotein gene, complete cds	73.1	73.1	100%	2e-10	100.00%	MK216695.1
Zika virus isolate AMA15 polyprotein gene, partial cds	73.1	73.1	100%	2e-10	100.00%	MK216694.1
Zika virus isolate AMA14 polyprotein gene, partial cds	73.1	73.1	100%	2e-10	100.00%	MK216693.1
Zika virus isolate AMA13 polyprotein gene, complete cds	73.1	73.1	100%	2e-10	100.00%	MK216692.1
Zika virus isolate AMA6 polyprotein gene, partial cds	73.1	73.1	100%	2e-10	100.00%	MK216691.1
Zika virus isolate AMA5 polyprotein gene, complete cds	73.1	73.1	100%	2e-10	100.00%	MK216688.1
Zika virus isolate AMA2 polyprotein gene, complete cds	73.1	73.1	100%	2e-10	100.00%	MK216687.1

Zika K39

CTGCTCCCAAGGAAGTAAAAAAGGGGGAGACCACAGATG

Descriptions | Graphic Summary | Alignments | **Taxonomy**

Reports | **Lineage** | Organism | Taxonomy

50 sequences selected

Organism	Blast Name	Score	Number of Hits	Description
Zika virus	viruses	73.1	51	Zika virus hits

Description	Max Score	Total Score	Query Cover	E value	Per. Ident	Accession
Zika virus isolate JXLHS1807 polyprotein gene, complete cds	73.1	73.1	100%	2e-10	100.00%	MK696551.1
Zika virus isolate 15098, complete genome	73.1	73.1	100%	2e-10	100.00%	MK566202.1
Zika virus PE243 polyprotein (POLY) gene, partial cds	73.1	73.1	100%	2e-10	100.00%	MT078742.1
Zika virus PE243 PE-innoculum polyprotein (POLY) gene, partial cds	73.1	73.1	100%	2e-10	100.00%	MT078741.1
Zika virus PE243 mouse-adapted 1 polyprotein (POLY) gene, partial cds	73.1	73.1	100%	2e-10	100.00%	MT078739.1
Zika virus isolate FLUR066 polyprotein gene, partial cds	73.1	73.1	100%	2e-10	100.00%	MK269361.1
Zika virus isolate Z02 polyprotein gene, partial cds	73.1	73.1	100%	2e-10	100.00%	MK216748.1
Zika virus isolate Z03 polyprotein gene, partial cds	73.1	73.1	100%	2e-10	100.00%	MK216747.1
Zika virus isolate AMA302 polyprotein gene, complete cds	73.1	73.1	100%	2e-10	100.00%	MK216745.1
Zika virus isolate AMA91 polyprotein gene, partial cds	73.1	73.1	100%	2e-10	100.00%	MK216744.1
Zika virus isolate AMA88 polyprotein gene, complete cds	73.1	73.1	100%	2e-10	100.00%	MK216743.1
Zika virus isolate AMA90 polyprotein gene, complete cds	73.1	73.1	100%	2e-10	100.00%	MK216742.1
Zika virus isolate AMA89 polyprotein gene, complete cds	73.1	73.1	100%	2e-10	100.00%	MK216741.1
Zika virus isolate AMA85 polyprotein gene, partial cds	73.1	73.1	100%	2e-10	100.00%	MK216740.1
Zika virus isolate AMA73 polyprotein gene, partial cds	73.1	73.1	100%	2e-10	100.00%	MK216739.1
Zika virus isolate AMA82 polyprotein gene, partial cds	73.1	73.1	100%	2e-10	100.00%	MK216738.1
Zika virus isolate AMA80 polyprotein gene, partial cds	73.1	73.1	100%	2e-10	100.00%	MK216737.1
Zika virus isolate AMA84 polyprotein gene, partial cds	73.1	73.1	100%	2e-10	100.00%	MK216736.1
Zika virus isolate AMA72 polyprotein gene, partial cds	73.1	73.1	100%	2e-10	100.00%	MK216735.1
Zika virus isolate AMA71 polyprotein gene, partial cds	73.1	73.1	100%	2e-10	100.00%	MK216734.1
Zika virus isolate AMA68 polyprotein gene, partial cds	73.1	73.1	100%	2e-10	100.00%	MK216733.1
Zika virus isolate AMA52 polyprotein gene, partial cds	73.1	73.1	100%	2e-10	100.00%	MK216732.1
Zika virus isolate AMA70 polyprotein gene, partial cds	73.1	73.1	100%	2e-10	100.00%	MK216731.1
Zika virus isolate AMA67 polyprotein gene, partial cds	73.1	73.1	100%	2e-10	100.00%	MK216730.1
Zika virus isolate AMA61 polyprotein gene, partial cds	73.1	73.1	100%	2e-10	100.00%	MK216729.1
Zika virus isolate AMA66 polyprotein gene, partial cds	73.1	73.1	100%	2e-10	100.00%	MK216728.1
Zika virus isolate AMA65 polyprotein gene, complete cds	73.1	73.1	100%	2e-10	100.00%	MK216727.1
Zika virus isolate AMA62 polyprotein gene, partial cds	73.1	73.1	100%	2e-10	100.00%	MK216726.1
Zika virus isolate AMA55 polyprotein gene, partial cds	73.1	73.1	100%	2e-10	100.00%	MK216725.1
Zika virus isolate AMA59 polyprotein gene, partial cds	73.1	73.1	100%	2e-10	100.00%	MK216724.1
Zika virus isolate AMA58 polyprotein gene, partial cds	73.1	73.1	100%	2e-10	100.00%	MK216722.1
Zika virus isolate AMA53 polyprotein gene, partial cds	73.1	73.1	100%	2e-10	100.00%	MK216721.1
Zika virus isolate AMA51 polyprotein gene, partial cds	73.1	73.1	100%	2e-10	100.00%	MK216720.1
Zika virus isolate AMA50 polyprotein gene, partial cds	73.1	73.1	100%	2e-10	100.00%	MK216719.1
Zika virus isolate AMA49 polyprotein gene, partial cds	73.1	73.1	100%	2e-10	100.00%	MK216718.1
Zika virus isolate AMA48 polyprotein gene, partial cds	73.1	73.1	100%	2e-10	100.00%	MK216717.1
Zika virus isolate AMA47 polyprotein gene, partial cds	73.1	73.1	100%	2e-10	100.00%	MK216716.1
Zika virus isolate AMA46 polyprotein gene, partial cds	73.1	73.1	100%	2e-10	100.00%	MK216715.1
Zika virus isolate AMA44 polyprotein gene, partial cds	73.1	73.1	100%	2e-10	100.00%	MK216714.1
Zika virus isolate AMA45 polyprotein gene, complete cds	73.1	73.1	100%	2e-10	100.00%	MK216713.1
Zika virus isolate AMA42 polyprotein gene, partial cds	73.1	73.1	100%	2e-10	100.00%	MK216712.1
Zika virus isolate AMA43 polyprotein gene, partial cds	73.1	73.1	100%	2e-10	100.00%	MK216711.1
Zika virus isolate AMA41 polyprotein gene, complete cds	73.1	73.1	100%	2e-10	100.00%	MK216710.1
Zika virus isolate AMA37 polyprotein gene, partial cds	73.1	73.1	100%	2e-10	100.00%	MK216709.1
Zika virus isolate AMA38 polyprotein gene, partial cds	73.1	73.1	100%	2e-10	100.00%	MK216708.1
Zika virus isolate AMA35 polyprotein gene, partial cds	73.1	73.1	100%	2e-10	100.00%	MK216707.1
Zika virus isolate AMA36 polyprotein gene, partial cds	73.1	73.1	100%	2e-10	100.00%	MK216706.1
Zika virus isolate AMA32 polyprotein gene, partial cds	73.1	73.1	100%	2e-10	100.00%	MK216705.1
Zika virus isolate AMA33 polyprotein gene, partial cds	73.1	73.1	100%	2e-10	100.00%	MK216704.1
Zika virus isolate AMA27 polyprotein gene, partial cds	73.1	73.1	100%	2e-10	100.00%	MK216703.1

Chikun K35

CACGTCGTGGGGGAGTACCTGGTACTTTCCCCGCA

Descriptions | Graphic Summary | Alignments | **Taxonomy**

Reports | **Lineage** | **Organism** | **Taxonomy**

100 sequences selected

Organism	Blast Name	Score	Number of Hits	Description
Chikungunya virus	viruses	65.8	100	Chikungunya virus hits

Description	Max Score	Total Score	Query Cover	E value	Per. Ident	Accession
Chikungunya virus strain Bol9 nonstructural polyprotein (NSP) and structural polyprotein (SF	65.8	65.8	100%	3e-08	100.00%	MT150100.1
Chikungunya virus strain Bol8 nonstructural polyprotein (NSP) and structural polyprotein (SF	65.8	65.8	100%	3e-08	100.00%	MT150099.1
Chikungunya virus strain Bol7 nonstructural polyprotein (NSP) and structural polyprotein (SF	65.8	65.8	100%	3e-08	100.00%	MT150098.1
Chikungunya virus strain Bol6 nonstructural polyprotein (NSP) and structural polyprotein (SF	65.8	65.8	100%	3e-08	100.00%	MT150097.1
Chikungunya virus strain Bol5 nonstructural polyprotein (NSP) and structural polyprotein (SF	65.8	65.8	100%	3e-08	100.00%	MT150096.1
Chikungunya virus strain Bol4 nonstructural polyprotein (NSP) and structural polyprotein (SF	65.8	65.8	100%	3e-08	100.00%	MT150095.1
Chikungunya virus strain Bol3 nonstructural polyprotein (NSP) and structural polyprotein (SF	65.8	65.8	100%	3e-08	100.00%	MT150094.1
Chikungunya virus strain Bol2 nonstructural polyprotein (NSP) and structural polyprotein (SF	65.8	65.8	100%	3e-08	100.00%	MT150093.1
Chikungunya virus strain Bol1 nonstructural polyprotein (NSP) and structural polyprotein (SF	65.8	65.8	100%	3e-08	100.00%	MT150092.1
Chikungunya virus ARUBA-15801654 RNA, complete genome	65.8	65.8	100%	3e-08	100.00%	LC500221.1
Chikungunya virus ARUBA-15801567 RNA, complete genome	65.8	65.8	100%	3e-08	100.00%	LC500220.1
Chikungunya virus ARUBA-15801358 RNA, complete genome	65.8	65.8	100%	3e-08	100.00%	LC500219.1
Chikungunya virus ARUBA-15801160 RNA, complete genome	65.8	65.8	100%	3e-08	100.00%	LC500218.1
Chikungunya virus ARUBA-15801136 RNA, complete genome	65.8	65.8	100%	3e-08	100.00%	LC500217.1
Chikungunya virus ARUBA-15801125 RNA, complete genome	65.8	65.8	100%	3e-08	100.00%	LC500216.1
Chikungunya virus ARUBA-15801056 RNA, complete genome	65.8	65.8	100%	3e-08	100.00%	LC500215.1
Chikungunya virus isolate KNIH/2013/707 non-structural protein gene, partial cds	65.8	65.8	100%	3e-08	100.00%	MF409047.1
Chikungunya virus isolate KNIH/2013/735 non-structural protein gene, partial cds	65.8	65.8	100%	3e-08	100.00%	MF409046.1
Chikungunya virus isolate KNIH/2014/513 non-structural protein gene, partial cds	65.8	65.8	100%	3e-08	100.00%	MF409045.1
Chikungunya virus isolate KNIH/2014/414 non-structural protein gene, partial cds	65.8	65.8	100%	3e-08	100.00%	MF409044.1
Chikungunya virus isolate KNIH/2013/756 non-structural protein gene, partial cds	65.8	65.8	100%	3e-08	100.00%	MF409043.1
Chikungunya virus strain OV-16, complete genome	65.8	65.8	100%	3e-08	100.00%	MH359142.1
Chikungunya virus strain OV-13, complete genome	65.8	65.8	100%	3e-08	100.00%	MH359141.1
Chikungunya virus strain Car-89, complete genome	65.8	65.8	100%	3e-08	100.00%	MH359140.1
Chikungunya virus strain OV-7, complete genome	65.8	65.8	100%	3e-08	100.00%	MH359139.1
Chikungunya virus strain Car-62, complete genome	65.8	65.8	100%	3e-08	100.00%	MH329304.1
Chikungunya virus strain Car-4, complete genome	65.8	65.8	100%	3e-08	100.00%	MH329303.1
Chikungunya virus strain Car-149, complete genome	65.8	65.8	100%	3e-08	100.00%	MH329302.1
Chikungunya virus strain Car-3, complete genome	65.8	65.8	100%	3e-08	100.00%	MH329301.1
Chikungunya virus strain Car-128, complete genome	65.8	65.8	100%	3e-08	100.00%	MH329300.1
Chikungunya virus strain INS-449125, complete genome	65.8	65.8	100%	3e-08	100.00%	MH329299.1
Chikungunya virus strain INS-449325, complete genome	65.8	65.8	100%	3e-08	100.00%	MH329298.1
Chikungunya virus strain INS- 477150, complete genome	65.8	65.8	100%	3e-08	100.00%	MH329297.1
Chikungunya virus strain M6, complete genome	65.8	65.8	100%	3e-08	100.00%	MH329296.1
Chikungunya virus strain P1, complete genome	65.8	65.8	100%	3e-08	100.00%	MH329295.1
Chikungunya virus strain OV-26, complete genome	65.8	65.8	100%	3e-08	100.00%	MH329294.1
Chikungunya virus strain Car-61, complete genome	65.8	65.8	100%	3e-08	100.00%	MH329293.1
Chikungunya virus, complete genome	65.8	65.8	100%	3e-08	100.00%	MH670649.1
Chikungunya virus isolate Chikungunya virus/H.sapiens-tc/Thailand/1962/AF15561, partial g	65.8	65.8	100%	3e-08	100.00%	MK028840.1
Chikungunya virus isolate Chikungunya virus/H.sapiens-tc/USA/1986/181-Clone 25, partial c	65.8	65.8	100%	3e-08	100.00%	MK028839.1
Chikungunya virus strain Homo sapiens/Haiti-11/2014, complete genome	65.8	65.8	100%	3e-08	100.00%	MG967666.1
Chikungunya virus isolate CH-R-1950, complete genome	65.8	65.8	100%	3e-08	100.00%	MG921596.1
Chikungunya virus strain SZ1239, complete genome	65.8	65.8	100%	3e-08	100.00%	MG664851.1
Chikungunya virus isolate ET2010, complete genome	65.8	65.8	100%	3e-08	100.00%	MF773565.1
Chikungunya virus isolate Philippines 2016, complete genome	65.8	65.8	100%	3e-08	100.00%	MF773564.1
Chikungunya virus isolate Philippines 2014, complete genome	65.8	65.8	100%	3e-08	100.00%	MF773563.1
Chikungunya virus isolate Kiribati 2015, complete genome	65.8	65.8	100%	3e-08	100.00%	MF773562.1
Chikungunya virus isolate Bali 2011, complete genome	65.8	65.8	100%	3e-08	100.00%	MF773561.1
Chikungunya virus isolate Caribbean 2014, complete genome	65.8	65.8	100%	3e-08	100.00%	MF773560.1
Chikungunya virus isolate Samoa 2014, complete genome	65.8	65.8	100%	3e-08	100.00%	MF773559.1

Chikun K35

GCTGTTGCAGCACTGATCCTAATCGTGGTGCTATG

Descriptions | Graphic Summary | Alignments | **Taxonomy**

Reports | **Lineage** | **Organism** | **Taxonomy**

100 sequences selected

Organism	Blast Name	Score	Number of Hits	Description
Chikungunya virus	viruses	65.8	100	Chikungunya virus hits

	Description	Max Score	Total Score	Query Cover	E value	Per. Ident	Accession
☑	Chikungunya virus strain Bol9 nonstructural polyprotein (NSP) and structural polyprotein (SP	65.8	65.8	100%	3e-08	100.00%	MT150100.1
☑	Chikungunya virus strain Bol8 nonstructural polyprotein (NSP) and structural polyprotein (SP	65.8	65.8	100%	3e-08	100.00%	MT150099.1
☑	Chikungunya virus strain Bol7 nonstructural polyprotein (NSP) and structural polyprotein (SP	65.8	65.8	100%	3e-08	100.00%	MT150098.1
☑	Chikungunya virus strain Bol6 nonstructural polyprotein (NSP) and structural polyprotein (SP	65.8	65.8	100%	3e-08	100.00%	MT150097.1
☑	Chikungunya virus strain Bol5 nonstructural polyprotein (NSP) and structural polyprotein (SP	65.8	65.8	100%	3e-08	100.00%	MT150096.1
☑	Chikungunya virus strain Bol4 nonstructural polyprotein (NSP) and structural polyprotein (SP	65.8	65.8	100%	3e-08	100.00%	MT150095.1
☑	Chikungunya virus strain Bol3 nonstructural polyprotein (NSP) and structural polyprotein (SP	65.8	65.8	100%	3e-08	100.00%	MT150094.1
☑	Chikungunya virus strain Bol2 nonstructural polyprotein (NSP) and structural polyprotein (SP	65.8	65.8	100%	3e-08	100.00%	MT150093.1
☑	Chikungunya virus strain Bol1 nonstructural polyprotein (NSP) and structural polyprotein (SP	65.8	65.8	100%	3e-08	100.00%	MT150092.1
☑	Chikungunya virus strain Asian isolate TA700_2015 Envelope protein 1 gene, partial cds	65.8	65.8	100%	3e-08	100.00%	MH500798.1
☑	Chikungunya virus strain Asian isolate TA711_2015 Envelope protein 1 gene, partial cds	65.8	65.8	100%	3e-08	100.00%	MH500797.1
☑	Chikungunya virus strain Asian isolate TA750_2015 Envelope protein 1 gene, partial cds	65.8	65.8	100%	3e-08	100.00%	MH500796.1
☑	Chikungunya virus strain Asian isolate TA712_2015 Envelope protein 1 gene, partial cds	65.8	65.8	100%	3e-08	100.00%	MH500795.1
☑	Chikungunya virus strain Asian isolate 15003_2015 Envelope protein 1 gene, partial cds	65.8	65.8	100%	3e-08	100.00%	MH500794.1
☑	Chikungunya virus ARUBA-15802650 RNA, complete genome	65.8	65.8	100%	3e-08	100.00%	LC500222.1
☑	Chikungunya virus ARUBA-15801654 RNA, complete genome	65.8	65.8	100%	3e-08	100.00%	LC500221.1
☑	Chikungunya virus ARUBA-15801567 RNA, complete genome	65.8	65.8	100%	3e-08	100.00%	LC500220.1
☑	Chikungunya virus ARUBA-15801358 RNA, complete genome	65.8	65.8	100%	3e-08	100.00%	LC500219.1
☑	Chikungunya virus ARUBA-15801160 RNA, complete genome	65.8	65.8	100%	3e-08	100.00%	LC500218.1
☑	Chikungunya virus ARUBA-15801136 RNA, complete genome	65.8	65.8	100%	3e-08	100.00%	LC500217.1
☑	Chikungunya virus ARUBA-15801125 RNA, complete genome	65.8	65.8	100%	3e-08	100.00%	LC500216.1
☑	Chikungunya virus ARUBA-15801056 RNA, complete genome	65.8	65.8	100%	3e-08	100.00%	LC500215.1
☑	Chikungunya virus strain Asian isolate TA679-2015 E2-6K-E1 gene, partial cds	65.8	65.8	100%	3e-08	100.00%	MK240311.1
☑	Chikungunya virus strain Asian isolate HU004-2015 E2-6K-E1 gene, partial cds	65.8	65.8	100%	3e-08	100.00%	MK240310.1
☑	Chikungunya virus isolate TC11 structural polyprotein gene, partial cds	65.8	65.8	100%	3e-08	100.00%	MH481900.1
☑	Chikungunya virus isolate MA003 structural polyprotein gene, partial cds	65.8	65.8	100%	3e-08	100.00%	MH481899.1
☑	Chikungunya virus isolate HU003 structural polyprotein gene, partial cds	65.8	65.8	100%	3e-08	100.00%	MH481898.1
☑	Chikungunya virus isolate CA002 structural polyprotein gene, partial cds	65.8	65.8	100%	3e-08	100.00%	MH481897.1
☑	Chikungunya virus isolate 755 structural polyprotein gene, partial cds	65.8	65.8	100%	3e-08	100.00%	MH481896.1
☑	Chikungunya virus isolate 750 structural polyprotein gene, partial cds	65.8	65.8	100%	3e-08	100.00%	MH481895.1
☑	Chikungunya virus isolate 725 structural polyprotein gene, partial cds	65.8	65.8	100%	3e-08	100.00%	MH481894.1
☑	Chikungunya virus isolate 712 structural polyprotein gene, partial cds	65.8	65.8	100%	3e-08	100.00%	MH481893.1
☑	Chikungunya virus isolate 711 structural polyprotein gene, partial cds	65.8	65.8	100%	3e-08	100.00%	MH481892.1
☑	Chikungunya virus isolate 700 structural polyprotein gene, partial cds	65.8	65.8	100%	3e-08	100.00%	MH481891.1
☑	Chikungunya virus isolate TC010 structural polyprotein gene, partial cds	65.8	65.8	100%	3e-08	100.00%	MH481890.1
☑	Chikungunya virus isolate 759 structural polyprotein gene, partial cds	65.8	65.8	100%	3e-08	100.00%	MH481889.1
☑	Chikungunya virus isolate 756 structural polyprotein gene, partial cds	65.8	65.8	100%	3e-08	100.00%	MH481888.1
☑	Chikungunya virus isolate 736 structural polyprotein gene, partial cds	65.8	65.8	100%	3e-08	100.00%	MH481887.1
☑	Chikungunya virus isolate 735c structural polyprotein gene, partial cds	65.8	65.8	100%	3e-08	100.00%	MH481886.1
☑	Chikungunya virus isolate 702 structural polyprotein gene, partial cds	65.8	65.8	100%	3e-08	100.00%	MH481885.1
☑	Chikungunya virus isolate 701 structural polyprotein gene, partial cds	65.8	65.8	100%	3e-08	100.00%	MH481884.1
☑	Chikungunya virus isolate 690 structural polyprotein gene, partial cds	65.8	65.8	100%	3e-08	100.00%	MH481883.1
☑	Chikungunya virus isolate 689 structural polyprotein gene, partial cds	65.8	65.8	100%	3e-08	100.00%	MH481882.1
☑	Chikungunya virus isolate 675 structural polyprotein gene, partial cds	65.8	65.8	100%	3e-08	100.00%	MH481881.1
☑	Chikungunya virus isolate KNIH/2014/513 envelope protein gene, partial cds	65.8	65.8	100%	3e-08	100.00%	MF409042.1
☑	Chikungunya virus isolate KNIH/2014/414 envelope protein gene, partial cds	65.8	65.8	100%	3e-08	100.00%	MF409041.1
☑	Chikungunya virus isolate KNIH/2013/756 envelope protein gene, partial cds	65.8	65.8	100%	3e-08	100.00%	MF409040.1
☑	Chikungunya virus isolate KNIH/2013/735 envelope protein gene, partial cds	65.8	65.8	100%	3e-08	100.00%	MF409039.1
☑	Chikungunya virus isolate KNIH/2013/707 envelope protein gene, partial cds	65.8	65.8	100%	3e-08	100.00%	MF409038.1
☑	Chikungunya virus strain OV-16, complete genome	65.8	65.8	100%	3e-08	100.00%	MH359142.1

Dengue K35

TGAGATACAAGAAAGCCACTTACGAGCCGGATGTT

Descriptions | Graphic Summary | Alignments | **Taxonomy**

Reports | **Lineage** | **Organism** | **Taxonomy**

50 sequences selected

Organism	Blast Name	Score	Number of Hits	Description
root			51	
. Flavivirus	viruses		48	
. . Dengue virus	viruses		10	
. . . Dengue virus 2	viruses	65.8	37	Dengue virus 2 hits
. . . Dengue virus 2 Thailand/16681/84	viruses	65.8	1	Dengue virus 2 Thailand/16681/84 hits
. . Dengue virus	viruses	65.8	10	Dengue virus hits
. synthetic construct	other sequences	65.8	3	synthetic construct hits

	Description	Max Score	Total Score	Query Cover	E value	Per. Ident	Accession
☑	Dengue virus 2 isolate 16681-2007, complete genome	65.8	65.8	100%	3e-08	100.00%	MK506263.1
☑	Dengue virus isolate Ser2_Thailand_nonBKK_Seq51 polyprotein gene, partial cds	65.8	65.8	100%	3e-08	100.00%	KY586699.1
☑	Dengue virus 2 strain PDK53, complete genome	65.8	65.8	100%	3e-08	100.00%	KU725664.1
☑	Dengue virus 2 strain 16681, complete genome	65.8	65.8	100%	3e-08	100.00%	KU725663.1
☑	Synthetic construct strain NS3hmin polyprotein gene, complete cds	65.8	65.8	100%	3e-08	100.00%	KP161066.1
☑	Synthetic construct strain Ehmin polyprotein gene, complete cds	65.8	65.8	100%	3e-08	100.00%	KP161065.1
☑	Synthetic construct strain D2syn polyprotein gene, complete cds	65.8	65.8	100%	3e-08	100.00%	KP161064.1
☑	Dengue virus 2 isolate DENV-2/TH/BID-V3357/1964, complete genome	65.8	65.8	100%	3e-08	100.00%	GQ868591.1
☑	Dengue virus type 2 polyprotein mRNA, complete cds	65.8	65.8	100%	3e-08	100.00%	U87412.1
☑	Dengue virus 2, complete genome	65.8	65.8	100%	3e-08	100.00%	NC_001474.2
☑	Dengue virus type 2 strain 16681 capsid protein (C) gene, matrix protein (M) gene, envelop	65.8	65.8	100%	3e-08	100.00%	M84727.1
☑	Dengue virus type 2 strain 16681-PDK53 capsid protein (C) gene, matrix protein (M) gene,	65.8	65.8	100%	3e-08	100.00%	M84728.1
☑	Dengue virus 2 isolate D15341, complete genome	60.2	60.2	100%	1e-06	97.14%	MN018345.1
☑	Dengue virus 2 isolate 19XN22111 polyprotein (POLY) gene, complete cds; and sfRNA1 lnc	60.2	60.2	100%	1e-06	97.14%	MN923122.1
☑	Dengue virus 2 isolate 19XN51040 polyprotein (POLY) gene, complete cds; and sfRNA1 lnc	60.2	60.2	100%	1e-06	97.14%	MN923120.1
☑	Dengue virus 2 isolate D2/China/GDmm/D15341/2015(Myanmar), complete genome	60.2	60.2	100%	1e-06	97.14%	MH827532.1
☑	Dengue virus 2 isolate DENV-2/China/YN/15DGR65(2015), complete genome	60.2	60.2	100%	1e-06	97.14%	KY672955.1
☑	Dengue virus 2 isolate DENV-2/China/YN/RL27(2013), complete genome	60.2	60.2	100%	1e-06	97.14%	KY672949.1
☑	Dengue virus 2 isolate DENV-2/China/YN/RL18(2013), complete genome	60.2	60.2	100%	1e-06	97.14%	KY672948.1
☑	Dengue virus 2 isolate DENV-2/China/YN/RL16(2013), complete genome	60.2	60.2	100%	1e-06	97.14%	KY672947.1
☑	Dengue virus 2 isolate DENV-2/China/YN/15DGR29(2015), complete genome	60.2	60.2	100%	1e-06	97.14%	KY672946.1
☑	Dengue virus 2 isolate DENV-2/China/YN/15DGR8(2015), complete genome	60.2	60.2	100%	1e-06	97.14%	KY672945.1
☑	Dengue virus 2 Th17-074DV2 gene for polyprotein, complete cds	60.2	60.2	100%	1e-06	97.14%	LC410188.1
☑	Dengue virus 2 Th17-061DV2 gene for polyprotein, complete cds	60.2	60.2	100%	1e-06	97.14%	LC410187.1
☑	Dengue virus 2 Th16-056DV2 gene for polyprotein, complete cds	60.2	60.2	100%	1e-06	97.14%	LC410186.1
☑	Dengue virus 2 Th16-035DV2 gene for polyprotein, complete cds	60.2	60.2	100%	1e-06	97.14%	LC410185.1
☑	Dengue virus 2 Th16-005DV2 gene for polyprotein, complete cds	60.2	60.2	100%	1e-06	97.14%	LC410184.1
☑	Dengue virus 2 isolate DENV-2/TH/1974, complete genome	60.2	60.2	100%	1e-06	97.14%	MK268692.1
☑	Dengue virus 2 isolate CNR_25326, complete genome	60.2	60.2	100%	1e-06	97.14%	MH888331.1
☑	Dengue virus 2 isolate YNPE2, complete genome	60.2	60.2	100%	1e-06	97.14%	MF459663.3
☑	Dengue virus 2 isolate UI17827 polyprotein gene, complete cds	60.2	60.2	100%	1e-06	97.14%	KY849768.1
☑	Dengue virus 2 isolate UI17564 polyprotein gene, complete cds	60.2	60.2	100%	1e-06	97.14%	KY849767.1
☑	Dengue virus 2 isolate UI16615 polyprotein gene, complete cds	60.2	60.2	100%	1e-06	97.14%	KY849766.1
☑	Dengue virus 2 isolate UI15067 polyprotein gene, complete cds	60.2	60.2	100%	1e-06	97.14%	KY849764.1
☑	Dengue virus 2 isolate UI18384 polyprotein gene, complete cds	60.2	60.2	100%	1e-06	97.14%	KY849763.1
☑	Dengue virus 2 isolate LNT713 polyprotein gene, complete cds	60.2	60.2	100%	1e-06	97.14%	KY849759.1
☑	Dengue virus 2 isolate SV397 polyprotein gene, complete cds	60.2	60.2	100%	1e-06	97.14%	KY849758.1
☑	Dengue virus 2 isolate SV576 polyprotein gene, complete cds	60.2	60.2	100%	1e-06	97.14%	KY849755.1
☑	Dengue virus 2 isolate LNT959 polyprotein gene, complete cds	60.2	60.2	100%	1e-06	97.14%	KY849754.1
☑	Dengue virus 2 isolate LNT866 polyprotein gene, complete cds	60.2	60.2	100%	1e-06	97.14%	KY849753.1
☑	Dengue virus 2 isolate LNT555 polyprotein gene, complete cds	60.2	60.2	100%	1e-06	97.14%	KY849752.1
☑	Dengue virus isolate Ser2_Thailand_Bangkok_Seq102 polyprotein gene, partial cds	60.2	60.2	100%	1e-06	97.14%	KY586698.1
☑	Dengue virus isolate Ser2_Thailand_Bangkok_Seq101 polyprotein gene, partial cds	60.2	60.2	100%	1e-06	97.14%	KY586697.1
☑	Dengue virus isolate Ser2_Thailand_Bangkok_Seq100 polyprotein gene, partial cds	60.2	60.2	100%	1e-06	97.14%	KY586696.1
☑	Dengue virus isolate Ser2_Thailand_nonBKK_Seq50 polyprotein gene, partial cds	60.2	60.2	100%	1e-06	97.14%	KY586695.1
☑	Dengue virus isolate Ser2_Thailand_Bangkok_Seq99 polyprotein gene, partial cds	60.2	60.2	100%	1e-06	97.14%	KY586694.1
☑	Dengue virus isolate Ser2_Thailand_Bangkok_Seq98 polyprotein gene, partial cds	60.2	60.2	100%	1e-06	97.14%	KY586693.1
☑	Dengue virus isolate Ser2_Thailand_Bangkok_Seq97 polyprotein gene, complete cds	60.2	60.2	100%	1e-06	97.14%	KY586692.1
☑	Dengue virus isolate Ser2_Thailand_Bangkok_Seq96 polyprotein gene, partial cds	60.2	60.2	100%	1e-06	97.14%	KY586691.1
☑	Dengue virus isolate Ser2_Thailand_nonBKK_Seq49 polyprotein gene, partial cds	60.2	60.2	100%	1e-06	97.14%	KY586690.1

Dengue K35

ACAGGAGACATCAAAGGAATCATGCAGGCAGGAAA

Descriptions | Graphic Summary | Alignments | **Taxonomy**

Reports | **Lineage** | **Organism** | **Taxonomy**

100 sequences selected

Organism	Blast Name	Score	Number of Hits	Description
Flavivirus	viruses		102	
. Dengue virus	viruses		3	
. . Dengue virus 2	viruses	65.8	99	Dengue virus 2 hits
. Dengue virus	viruses	65.8	3	Dengue virus hits

	Description	Max Score	Total Score	Query Cover	E value	Per. Ident	Accession
☑	Dengue virus 2 isolate AFI MDH 232 polyprotein (POLY) gene, partial cds	65.8	65.8	100%	3e-08	100.00%	MN335247.1
☑	Dengue virus 2 isolate AFI MDH 244 polyprotein (POLY) gene, partial cds	65.8	65.8	100%	3e-08	100.00%	MN335246.1
☑	Dengue virus 2 isolate AFI MDH 240 polyprotein (POLY) gene, partial cds	65.8	65.8	100%	3e-08	100.00%	MN335245.1
☑	Dengue virus 2 isolate AFI MDH 243 polyprotein (POLY) gene, partial cds	65.8	65.8	100%	3e-08	100.00%	MN335244.1
☑	Dengue virus 2 isolate D151665, complete genome	65.8	65.8	100%	3e-08	100.00%	MN018366.1
☑	Dengue virus 2 isolate D16033, complete genome	65.8	65.8	100%	3e-08	100.00%	MN018348.1
☑	Dengue virus 2 isolate D16158, complete genome	65.8	65.8	100%	3e-08	100.00%	MN018347.1
☑	Dengue virus 2 isolate D16005, complete genome	65.8	65.8	100%	3e-08	100.00%	MN018346.1
☑	Dengue virus 2 isolate D15341, complete genome	65.8	65.8	100%	3e-08	100.00%	MN018345.1
☑	Dengue virus 2 isolate D16032, complete genome	65.8	65.8	100%	3e-08	100.00%	MN018340.1
☑	Dengue virus 2 isolate 19XN22111 polyprotein (POLY) gene, complete cds; and sfRNA1 lncf	65.8	65.8	100%	3e-08	100.00%	MN923122.1
☑	Dengue virus 2 isolate 19XN51040 polyprotein (POLY) gene, complete cds; and sfRNA1 lncl	65.8	65.8	100%	3e-08	100.00%	MN923120.1
☑	Dengue virus 2 isolate 19XN50506 polyprotein (POLY) gene, complete cds; and sfRNA1 lncl	65.8	65.8	100%	3e-08	100.00%	MN923115.1
☑	Dengue virus 2 isolate 19XN28420 polyprotein (POLY) gene, complete cds; and sfRNA1 lncl	65.8	65.8	100%	3e-08	100.00%	MN923114.1
☑	Dengue virus 2 isolate 19XN25699 polyprotein (POLY) gene, complete cds; and sfRNA1 lncl	65.8	65.8	100%	3e-08	100.00%	MN923113.1
☑	Dengue virus 2 isolate 19XN21330 polyprotein (POLY) gene, complete cds; and sfRNA1 lncl	65.8	65.8	100%	3e-08	100.00%	MN923111.1
☑	Dengue virus 2 isolate 19XN17542 polyprotein (POLY) gene, complete cds; and sfRNA1 lncl	65.8	65.8	100%	3e-08	100.00%	MN923109.1
☑	Dengue virus 2 isolate MCVR7291AD/2018, complete genome	65.8	65.8	100%	3e-08	100.00%	MH891770.1
☑	Dengue virus 2 isolate R1 J15, complete genome	65.8	65.8	100%	3e-08	100.00%	MG592698.1
☑	Dengue virus 2 isolate D2/China/GDqy/GD16158/2016, complete genome	65.8	65.8	100%	3e-08	100.00%	MH827543.1
☑	Dengue virus 2 isolate D2/China/GDsd/GD16033/2016, complete genome	65.8	65.8	100%	3e-08	100.00%	MH827541.1
☑	Dengue virus 2 isolate D2/China/GDfs/GD16032/2016, complete genome	65.8	65.8	100%	3e-08	100.00%	MH827540.1
☑	Dengue virus 2 isolate D2/China/GD /GD151665/2015, complete genome	65.8	65.8	100%	3e-08	100.00%	MH827538.1
☑	Dengue virus 2 isolate D2/China/GDmm/D16005/2016(Malaysia), complete genome	65.8	65.8	100%	3e-08	100.00%	MH827536.1
☑	Dengue virus 2 isolate D2/China/GDmm/D15341/2015(Myanmar), complete genome	65.8	65.8	100%	3e-08	100.00%	MH827532.1
☑	Dengue virus 2 isolate Henan201803 polyprotein, nonstructural protein NS1 region, (POLY)	65.8	65.8	100%	3e-08	100.00%	MK969680.1
☑	Dengue virus 2 isolate 2018-GZ-462 nonstructural protein 1 gene, partial cds	65.8	65.8	100%	3e-08	100.00%	MK140754.1
☑	Dengue virus 2 isolate 43253 polyprotein (POLY) gene, complete cds	65.8	65.8	100%	3e-08	100.00%	MK629885.1
☑	Dengue virus 2 isolate GZ18XN20079 polyprotein (POLY) gene, complete cds	65.8	65.8	100%	3e-08	100.00%	MK578533.1
☑	Dengue virus 2 isolate GZ16XN10007 polyprotein (POLY) gene, complete cds	65.8	65.8	100%	3e-08	100.00%	MK578532.1
☑	Dengue virus 2 isolate GZ18XN60367 polyprotein (POLY) gene, complete cds	65.8	65.8	100%	3e-08	100.00%	MK564488.1
☑	Dengue virus 2 isolate GZ16XN18674 polyprotein (POLY) gene, complete cds	65.8	65.8	100%	3e-08	100.00%	MK564479.1
☑	Dengue virus 2 isolate AO-1 polyprotein gene, partial cds	65.8	65.8	100%	3e-08	100.00%	MH460898.1
☑	Dengue virus 2 isolate C6L P56, complete genome	65.8	65.8	100%	3e-08	100.00%	MH613986.1
☑	Dengue virus 2 isolate C6L P30, complete genome	65.8	65.8	100%	3e-08	100.00%	MH613985.1
☑	Dengue virus 2 isolate C6L 48h, complete genome	65.8	65.8	100%	3e-08	100.00%	MH613984.1
☑	Dengue virus 2 isolate ngc-2007, complete genome	65.8	65.8	100%	3e-08	100.00%	MK506264.1
☑	Dengue virus 2 isolate 16681-2007, complete genome	65.8	65.8	100%	3e-08	100.00%	MK506263.1
☑	Dengue virus 2 isolate DENV-2/China/YN/15DGR65(2015), complete genome	65.8	65.8	100%	3e-08	100.00%	KY672955.1
☑	Dengue virus 2 isolate DENV-2/China/YN/RL18(2013), complete genome	65.8	65.8	100%	3e-08	100.00%	KY672948.1
☑	Dengue virus 2 isolate DENV-2/China/YN/15DGR29(2015), complete genome	65.8	65.8	100%	3e-08	100.00%	KY672946.1
☑	Dengue virus 2 isolate DENV-2/China/YN/15DGR8(2015), complete genome	65.8	65.8	100%	3e-08	100.00%	KY672945.1
☑	Dengue virus 2 Th17-074DV2 gene for polyprotein, complete cds	65.8	65.8	100%	3e-08	100.00%	LC410188.1
☑	Dengue virus 2 Th17-061DV2 gene for polyprotein, complete cds	65.8	65.8	100%	3e-08	100.00%	LC410187.1
☑	Dengue virus 2 Th16-056DV2 gene for polyprotein, complete cds	65.8	65.8	100%	3e-08	100.00%	LC410186.1
☑	Dengue virus 2 Th16-035DV2 gene for polyprotein, complete cds	65.8	65.8	100%	3e-08	100.00%	LC410185.1
☑	Dengue virus 2 Th16-005DV2 gene for polyprotein, complete cds	65.8	65.8	100%	3e-08	100.00%	LC410184.1
☑	Dengue virus 2 strain UoH 583351, complete genome	65.8	65.8	100%	3e-08	100.00%	MG560144.1
☑	Dengue virus 2 strain UoH 58620, complete genome	65.8	65.8	100%	3e-08	100.00%	MG560143.1
☑	Dengue virus 2 isolate DENV2/KLF/008/2014 polyprotein gene, partial cds	65.8	65.8	100%	3e-08	100.00%	MH456899.1

HIV K27

GGGACAATTGGAGAAGTGAATTATATA

Descriptions | Graphic Summary | Alignments | **Taxonomy**

Reports | **Lineage** | **Organism** | **Taxonomy**

100 sequences selected

Organism	Blast Name	Score	Number of Hits	Description
Human immunodeficiency virus 1	viruses	54.0	100	Human immunodeficiency virus 1 hits

	Description	Max Score	Total Score	Query Cover	E value	Per. Ident	Accession
☑	HIV-1 isolate S146_S2_H5 from Malawi envelope glycoprotein (env) gene, complete cds	54.0	54.0	100%	9e-05	100.00%	MT227491.1
☑	HIV-1 isolate S146_B2_D10 from Malawi envelope glycoprotein (env) gene, complete cds	54.0	54.0	100%	9e-05	100.00%	MT227470.1
☑	HIV-1 isolate S146_B2_F8b from Malawi envelope glycoprotein (env) gene, complete cds	54.0	54.0	100%	9e-05	100.00%	MT227469.1
☑	HIV-1 isolate S146_B2_A3 from Malawi envelope glycoprotein (env) gene, complete cds	54.0	54.0	100%	9e-05	100.00%	MT227468.1
☑	HIV-1 isolate S146_B2_G10b from Malawi envelope glycoprotein (env) gene, complete cds	54.0	54.0	100%	9e-05	100.00%	MT227467.1
☑	HIV-1 isolate S146_B2_F9b from Malawi envelope glycoprotein (env) gene, complete cds	54.0	54.0	100%	9e-05	100.00%	MT227466.1
☑	HIV-1 isolate S146_S2_F7 from Malawi envelope glycoprotein (env) gene, complete cds	54.0	54.0	100%	9e-05	100.00%	MT227465.1
☑	HIV-1 isolate S146_B2_E7 from Malawi envelope glycoprotein (env) gene, complete cds	54.0	54.0	100%	9e-05	100.00%	MT227464.1
☑	HIV-1 isolate C061_S3_G11 from Malawi envelope glycoprotein (env) gene, complete cds	54.0	54.0	100%	9e-05	100.00%	MT227463.1
☑	HIV-1 isolate C061_S2_H8 from Malawi envelope glycoprotein (env) gene, complete cds	54.0	54.0	100%	9e-05	100.00%	MT227462.1
☑	HIV-1 isolate C061_S3_F5 from Malawi envelope glycoprotein (env) gene, complete cds	54.0	54.0	100%	9e-05	100.00%	MT227461.1
☑	HIV-1 isolate C061_B2_E2 from Malawi envelope glycoprotein (env) gene, complete cds	54.0	54.0	100%	9e-05	100.00%	MT227460.1
☑	HIV-1 isolate C061_S3_G9 from Malawi envelope glycoprotein (env) gene, complete cds	54.0	54.0	100%	9e-05	100.00%	MT227459.1
☑	HIV-1 isolate C061_S3_E7 from Malawi envelope glycoprotein (env) gene, complete cds	54.0	54.0	100%	9e-05	100.00%	MT227458.1
☑	HIV-1 isolate C061_S2_B7 from Malawi envelope glycoprotein (env) gene, complete cds	54.0	54.0	100%	9e-05	100.00%	MT227457.1
☑	HIV-1 isolate C061_S2_E9 from Malawi envelope glycoprotein (env) gene, complete cds	54.0	54.0	100%	9e-05	100.00%	MT227456.1
☑	HIV-1 isolate C061_S2_A7 from Malawi envelope glycoprotein (env) gene, complete cds	54.0	54.0	100%	9e-05	100.00%	MT227455.1
☑	HIV-1 isolate C061_S2_C8 from Malawi envelope glycoprotein (env) gene, complete cds	54.0	54.0	100%	9e-05	100.00%	MT227454.1
☑	HIV-1 isolate C061_S2_B11 from Malawi envelope glycoprotein (env) gene, complete cds	54.0	54.0	100%	9e-05	100.00%	MT227453.1
☑	HIV-1 isolate C061_S3_H5 from Malawi envelope glycoprotein (env) gene, complete cds	54.0	54.0	100%	9e-05	100.00%	MT227452.1
☑	HIV-1 isolate C061_S3_B1 from Malawi envelope glycoprotein (env) gene, complete cds	54.0	54.0	100%	9e-05	100.00%	MT227451.1
☑	HIV-1 isolate C061_S2_F11 from Malawi envelope glycoprotein (env) gene, complete cds	54.0	54.0	100%	9e-05	100.00%	MT227450.1
☑	HIV-1 isolate C061_S3_D11 from Malawi envelope glycoprotein (env) gene, complete cds	54.0	54.0	100%	9e-05	100.00%	MT227449.1
☑	HIV-1 isolate C061_S3_B4 from Malawi envelope glycoprotein (env) gene, complete cds	54.0	54.0	100%	9e-05	100.00%	MT227448.1
☑	HIV-1 isolate C061_S2_A12 from Malawi envelope glycoprotein (env) gene, complete cds	54.0	54.0	100%	9e-05	100.00%	MT227447.1
☑	HIV-1 isolate C061_S2_A9 from Malawi envelope glycoprotein (env) gene, complete cds	54.0	54.0	100%	9e-05	100.00%	MT227446.1
☑	HIV-1 isolate C061_S3_B2 from Malawi envelope glycoprotein (env) gene, complete cds	54.0	54.0	100%	9e-05	100.00%	MT227445.1
☑	HIV-1 isolate C061_S3_F12 from Malawi envelope glycoprotein (env) gene, complete cds	54.0	54.0	100%	9e-05	100.00%	MT227444.1
☑	HIV-1 isolate C061_S3_A5 from Malawi envelope glycoprotein (env) gene, complete cds	54.0	54.0	100%	9e-05	100.00%	MT227443.1
☑	HIV-1 isolate C061_S3_D7 from Malawi envelope glycoprotein (env) gene, complete cds	54.0	54.0	100%	9e-05	100.00%	MT227442.1
☑	HIV-1 isolate C061_S3_A2 from Malawi envelope glycoprotein (env) gene, complete cds	54.0	54.0	100%	9e-05	100.00%	MT227441.1
☑	HIV-1 isolate C061_S2_A11 from Malawi envelope glycoprotein (env) gene, complete cds	54.0	54.0	100%	9e-05	100.00%	MT227440.1
☑	HIV-1 isolate C061_S2_B12 from Malawi envelope glycoprotein (env) gene, complete cds	54.0	54.0	100%	9e-05	100.00%	MT227438.1
☑	HIV-1 isolate C061_S3_B12 from Malawi envelope glycoprotein (env) gene, complete cds	54.0	54.0	100%	9e-05	100.00%	MT227437.1
☑	HIV-1 isolate C061_S2_C12 from Malawi envelope glycoprotein (env) gene, complete cds	54.0	54.0	100%	9e-05	100.00%	MT227436.1
☑	HIV-1 isolate C061_S2_B10 from Malawi envelope glycoprotein (env) gene, complete cds	54.0	54.0	100%	9e-05	100.00%	MT227435.1
☑	HIV-1 isolate C061_B2_H2 from Malawi envelope glycoprotein (env) gene, complete cds	54.0	54.0	100%	9e-05	100.00%	MT227434.1
☑	HIV-1 isolate C061_B2_A3 from Malawi envelope glycoprotein (env) gene, complete cds	54.0	54.0	100%	9e-05	100.00%	MT227433.1
☑	HIV-1 isolate S031_S2_F7 from Malawi envelope glycoprotein (env) gene, complete cds	54.0	54.0	100%	9e-05	100.00%	MT227414.1
☑	HIV-1 isolate S031_S2_B10 from Malawi envelope glycoprotein (env) gene, complete cds	54.0	54.0	100%	9e-05	100.00%	MT227413.1
☑	HIV-1 isolate S101_S101_S1_H4 from Malawi envelope glycoprotein (env) gene, complete c	54.0	54.0	100%	9e-05	100.00%	MT227411.1
☑	HIV-1 isolate S101_B3_F4 from Malawi envelope glycoprotein (env) gene, complete cds	54.0	54.0	100%	9e-05	100.00%	MT227410.1
☑	HIV-1 isolate S101_S101_B1_C4 from Malawi envelope glycoprotein (env) gene, complete c	54.0	54.0	100%	9e-05	100.00%	MT227409.1
☑	HIV-1 isolate S101_B3_H7 from Malawi envelope glycoprotein (env) gene, complete cds	54.0	54.0	100%	9e-05	100.00%	MT227408.1
☑	HIV-1 isolate S101_S101_B1_B1 from Malawi envelope glycoprotein (env) gene, complete c	54.0	54.0	100%	9e-05	100.00%	MT227406.1
☑	HIV-1 isolate S101_S101_S1_E2 from Malawi envelope glycoprotein (env) gene, complete c	54.0	54.0	100%	9e-05	100.00%	MT227405.1
☑	HIV-1 isolate S101_B3_A3 from Malawi envelope glycoprotein (env) gene, complete cds	54.0	54.0	100%	9e-05	100.00%	MT227404.1
☑	HIV-1 isolate S101_B3_H6 from Malawi envelope glycoprotein (env) gene, complete cds	54.0	54.0	100%	9e-05	100.00%	MT227403.1
☑	HIV-1 isolate S101_B3_G1 from Malawi envelope glycoprotein (env) gene, complete cds	54.0	54.0	100%	9e-05	100.00%	MT227402.1
☑	HIV-1 isolate S101_S101_S1_B6 from Malawi envelope glycoprotein (env) gene, complete c	54.0	54.0	100%	9e-05	100.00%	MT227401.1

HIV K27

TGATGATCTGTAGTGCTACAGAAAAAT

Descriptions | Graphic Summary | Alignments | **Taxonomy**

Reports | **Lineage** | **Organism** | **Taxonomy**

100 sequences selected

Organism	Blast Name	Score	Number of Hits	Description
root			100	
. Human immunodeficiency virus 1	viruses	54.0	86	Human immunodeficiency virus 1 hits
. synthetic construct	other sequences	54.0	14	synthetic construct hits

Description	Max Score	Total Score	Query Cover	E value	Per. Ident	Accession
Mutant HIV-1 isolate J-Lat10.6 HIV, complete genome	54.0	54.0	100%	9e-05	100.00%	MN989412.1
HIV-1 isolate 1408 TCM 48 from USA defective genome	54.0	54.0	100%	9e-05	100.00%	MN467309.1
HIV-1 isolate ACH2-NFLMDA13 B1 from USA, complete genome	54.0	54.0	100%	9e-05	100.00%	MN691959.1
Synthetic construct strain NL4-3 env611-1014CpG, complete sequence	54.0	54.0	100%	9e-05	100.00%	MN685351.1
Synthetic construct strain NL4-3 pol795-1386CpG, complete sequence	54.0	54.0	100%	9e-05	100.00%	MN685349.1
Synthetic construct strain NL4-3 gag694-1206CpG, complete sequence	54.0	54.0	100%	9e-05	100.00%	MN685348.1
Synthetic construct strain NL4-3 gag22-1185CpG, complete sequence	54.0	54.0	100%	9e-05	100.00%	MN685347.1
Synthetic construct strain NL4-3 gag660-1185CpG, complete sequence	54.0	54.0	100%	9e-05	100.00%	MN685346.1
Synthetic construct strain NL4-3 gag22-651CpG, complete sequence	54.0	54.0	100%	9e-05	100.00%	MN685345.1
Synthetic construct strain NL4-3 gag22-378CM-5nt-CpG, complete sequence	54.0	54.0	100%	9e-05	100.00%	MN685344.1
Synthetic construct strain NL4-3 gag22-378DC, complete sequence	54.0	54.0	100%	9e-05	100.00%	MN685343.1
Synthetic construct strain NL4-3 gag22-378CpG, complete sequence	54.0	54.0	100%	9e-05	100.00%	MN685342.1
Synthetic construct strain NL4-3 gag22-378CM-no-CpG, complete sequence	54.0	54.0	100%	9e-05	100.00%	MN685341.1
Synthetic construct strain NL4-3 gag22-378CM, complete sequence	54.0	54.0	100%	9e-05	100.00%	MN685340.1
Synthetic construct strain NL4-3 gag22-261CM, complete sequence	54.0	54.0	100%	9e-05	100.00%	MN685339.1
Synthetic construct strain NL4-3 gag22-165CM, complete sequence	54.0	54.0	100%	9e-05	100.00%	MN685338.1
Synthetic construct strain NL4-3, complete sequence	54.0	54.0	100%	9e-05	100.00%	MN685337.1
HIV-1 isolate CB from Belgium, complete genome	54.0	54.0	100%	9e-05	100.00%	MN486024.1
HIV-1 clone PL63-128-2 from Spain envelope glycoprotein (env) gene, partial cds	54.0	54.0	100%	9e-05	100.00%	MN395385.1
HIV-1 isolate 2669.PBMC.CAD.03142016.ENV-Seq14 from USA envelope glycoprotein (env	54.0	54.0	100%	9e-05	100.00%	MK148577.1
HIV-1 isolate 2115-DRp-4 from USA nonfunctional gag protein (gag) gene, complete sequen	54.0	54.0	100%	9e-05	100.00%	MH843864.1
HIV-1 isolate 2115-DRp-27 from USA gag protein (gag) gene, complete cds; pol protein (pol)	54.0	54.0	100%	9e-05	100.00%	MH843860.1
HIV-1 isolate 2115-DRp-24 from USA gag protein (gag) gene, complete cds; pol protein (pol)	54.0	54.0	100%	9e-05	100.00%	MH843857.1
HIV-1 isolate 2115-DRp-10 from USA nonfunctional gag protein (gag) gene, complete seque	54.0	54.0	100%	9e-05	100.00%	MH843852.1
HIV-1 isolate 2115-DRp-1 from USA gag protein (gag) gene, complete cds; pol protein (pol) c	54.0	54.0	100%	9e-05	100.00%	MH843851.1
HIV-1 isolate 2115-DRn-8 from USA nonfunctional gag protein (gag) gene, complete sequen	54.0	54.0	100%	9e-05	100.00%	MH843850.1
HIV-1 isolate 2115-DRn-33 from USA nonfunctional gag protein (gag) gene, complete seque	54.0	54.0	100%	9e-05	100.00%	MH843843.1
HIV-1 isolate 2115-DRn-3 from USA nonfunctional gag protein (gag) gene, complete sequen	54.0	54.0	100%	9e-05	100.00%	MH843840.1
HIV-1 isolate 2115-DRn-2 from USA nonfunctional gag protein (gag) gene, complete sequen	54.0	54.0	100%	9e-05	100.00%	MH843830.1
HIV-1 isolate 2115-DRn-19 from USA nonfunctional gag protein (gag) gene, complete seque	54.0	54.0	100%	9e-05	100.00%	MH843829.1
HIV-1 isolate 2115-DRn-11 from USA nonfunctional gag protein (gag) gene, complete seque	54.0	54.0	100%	9e-05	100.00%	MH843823.1
HIV-1 isolate 2115-DRn-21 from USA nonfunctional pol protein (pol) gene, partial sequence;	54.0	54.0	100%	9e-05	100.00%	MH843832.1
HIV-1 isolate 2115-DRn-12 from USA nonfunctional gag protein (gag) gene, complete seque	54.0	54.0	100%	9e-05	100.00%	MH843824.1
HIV-1 isolate 9GW09P1D5 from USA defective genome genomic sequence	54.0	54.0	100%	9e-05	100.00%	MK385585.1
HIV-1 isolate 9BPGW2015P5A8 from USA defective genome genomic sequence	54.0	54.0	100%	9e-05	100.00%	MK385584.1
HIV-1 isolate 98GW2011P7C12 from USA defective genome genomic sequence	54.0	54.0	100%	9e-05	100.00%	MK385576.1
HIV-1 isolate 96GW07P5D8 from USA defective genome genomic sequence	54.0	54.0	100%	9e-05	100.00%	MK385560.1
HIV-1 isolate 94BPGW2015P8F5 from USA defective genome genomic sequence	54.0	54.0	100%	9e-05	100.00%	MK385548.1
HIV-1 isolate 93BPGW2015P8F4 from USA defective genome genomic sequence	54.0	54.0	100%	9e-05	100.00%	MK385543.1
HIV-1 isolate 92GW11P5D12 from USA defective genome genomic sequence	54.0	54.0	100%	9e-05	100.00%	MK385539.1
HIV-1 isolate 92BPGW2015P8E12 from USA defective genome genomic sequence	54.0	54.0	100%	9e-05	100.00%	MK385538.1
HIV-1 isolate 91BPGW2015P8D10 from USA defective genome genomic sequence	54.0	54.0	100%	9e-05	100.00%	MK385534.1
HIV-1 isolate 90GW11P5D3 from USA defective genome genomic sequence	54.0	54.0	100%	9e-05	100.00%	MK385530.1
HIV-1 isolate 90BPGW2015P8D2 from USA defective genome genomic sequence	54.0	54.0	100%	9e-05	100.00%	MK385528.1
HIV-1 isolate 8JL10P1B8 from USA defective genome genomic sequence	54.0	54.0	100%	9e-05	100.00%	MK385523.1
HIV-1 isolate 8GW14P1D11 from USA defective genome genomic sequence	54.0	54.0	100%	9e-05	100.00%	MK385520.1
HIV-1 isolate 8GW11P1D11 from USA defective genome genomic sequence	54.0	54.0	100%	9e-05	100.00%	MK385519.1
HIV-1 isolate 8GW09P1D4 from USA defective genome genomic sequence	54.0	54.0	100%	9e-05	100.00%	MK385518.1
HIV-1 isolate 8BPGW2015P4G8 from USA defective genome genomic sequence	54.0	54.0	100%	9e-05	100.00%	MK385516.1
HIV-1 isolate 89GW07P5B9 from USA defective genome genomic sequence	54.0	54.0	100%	9e-05	100.00%	MK385511.1

Influenza K23

GGACAAACTTTATGGAGTAAAAT

Descriptions | Graphic Summary | Alignments | **Taxonomy**

Reports | **Lineage** | Organism | Taxonomy

100 sequences selected

Organism	Blast Name	Score	Number of Hits
root			145
. Alphainfluenzavirus	viruses		144

	Description	Max Score	Total Score	Query Cover	E value	Per. Ident	Accession
☑	Influenza A virus (A/swine/China/AV1523PB2-1.seq/2011(H1N1)) segment 1 polymerase PB2	46.1	46.1	100%	0.011	100.00%	MN700049.1
☑	Influenza A virus (A/Avian/Iran/38AMA/2019(H5N3)) segment 1 polymerase PB2 (PB2) gene,	46.1	46.1	100%	0.011	100.00%	MN453436.1
☑	Influenza A virus (A/Basel/USB002464.1/2017(H3N2)) segment 1 polymerase PB2 (PB2) ger	46.1	46.1	100%	0.011	100.00%	MN304274.1
☑	Influenza A virus (A/USA/rWSN/2000(H1N1)) segment 1 polymerase PB2 (PB2) gene, compl	46.1	46.1	100%	0.011	100.00%	MN220699.1
☑	Influenza A virus (A/Moscow/rWSN/2016(H1N1)) clone mrWSN_M1_126S segment 1 polyme	46.1	46.1	100%	0.011	100.00%	MN220691.1
☑	Influenza A virus (A/Puerto Rico/8/1934(H1N1)) segment 1 polymerase PB2 (PB2) gene, com	46.1	46.1	100%	0.011	100.00%	MH785018.1
☑	Influenza A virus (A/Ohio/7248/2018(H3N2)) segment 1 polymerase PB2 (PB2) gene, comple	46.1	46.1	100%	0.011	100.00%	MK401876.1
☑	Influenza A virus (A/swine/Michoacan/MichDMZC10/2015(H1N1)) segment 1 polymerase PB	46.1	46.1	100%	0.011	100.00%	MH006707.1
☑	Influenza A virus (A/PR/8_RGCDC-4.6/34(H1N1)) segment 1 polymerase PB2 (PB2) gene, c	46.1	46.1	100%	0.011	100.00%	MH201221.1
☑	Influenza A virus (A/Texas/292/2017(H3N2)) polymerase PB2 (PB2) gene, complete cds	46.1	46.1	100%	0.011	100.00%	CY260395.1
☑	Influenza A virus (A/WSN/1933(H1N1)) viral cRNA, segment 1, complete sequence	46.1	46.1	100%	0.011	100.00%	LC333182.1
☑	Influenza A virus (A/guineafowl/Hong Kong/WF10_CIP046_RGAO032/1999(H9N1)) clone AC	46.1	46.1	100%	0.011	100.00%	KX859433.1
☑	Influenza A virus (A/turkey/OH/313053_CIP046_RGJS71/2004(H3N2)) clone JS71_S17 segr	46.1	46.1	100%	0.011	100.00%	KX859432.1
☑	Influenza A virus (A/guineafowl/Hong Kong/WF10_CIP046_RGAO032/1999(H9N1)) clone AC	46.1	46.1	100%	0.011	100.00%	KX859431.1
☑	Influenza A virus (A/guineafowl/Hong Kong/WF10_CIP046_RGAO032/1999(H9N1)) clone AC	46.1	46.1	100%	0.011	100.00%	KX859356.1
☑	Influenza A virus (A/guineafowl/Hong Kong/WF10_CIP046_RGAO032/1999(H9N1)) clone AC	46.1	46.1	100%	0.011	100.00%	KX859343.1
☑	Influenza A virus (A/Puerto Rico/8/1934(H1N1)) RNA, segment 1, complete sequence	46.1	46.1	100%	0.011	100.00%	LC120388.1
☑	Influenza A virus (A/Missouri/11/2015(H3N2)) segment 1 polymerase PB2 (PB2) gene, compl	46.1	46.1	100%	0.011	100.00%	KT836383.1
☑	Influenza A virus (A/Missouri/09/2015(H3N2)) segment 1 polymerase PB2 (PB2) gene, comp	46.1	46.1	100%	0.011	100.00%	KT836217.1
☑	Influenza A virus (A/Puerto Rico/8HY/1934(H1N1)) segment 1 polymerase PB2 (PB2) gene,	46.1	46.1	100%	0.011	100.00%	KT314339.1
☑	Influenza A virus (A/reassortant/IgYRP16(California/07/2004 x Puerto Rico/8/1934)(H3N2)) s	46.1	46.1	100%	0.011	100.00%	JF690257.2
☑	Influenza A virus (A/mink/Shandong/F10/2013(H9N2)) segment 1 polymerase PB2 (PB2) ger	46.1	46.1	100%	0.011	100.00%	KM576118.1
☑	Influenza A virus (A/NWS/1933(H1N1)) pb2 gene for polymerase basic 2, complete cds, strai	46.1	46.1	100%	0.011	100.00%	AB981587.1
☑	Influenza A virus (A/reassortant/X-139(X-31B x New Caledonia/20/1999)(H1N1)) segment 1	46.1	46.1	100%	0.011	100.00%	KJ942815.1
☑	Influenza A virus (A/reassortant/NYMC X-223A(A/Puerto Rico/8/1934 x A/Texas/50/2012)(H3	46.1	46.1	100%	0.011	100.00%	KJ942791.1
☑	Influenza A virus (A/reassortant/X-175C(Uruguay/716/2007 x Puerto Rico/8/1934)(H3N2)) se	46.1	46.1	100%	0.011	100.00%	KJ942775.1
☑	Influenza A virus (A/reassortant/NYMC X-203(A/Puerto Rico/8/1934 x A/Minnesota/11/2010)(	46.1	46.1	100%	0.011	100.00%	KJ942767.1
☑	Influenza A virus (A/reassortant/NYMC X-197(Brisbane/11/2010 x Puerto Rico/8/1934)(H3N2	46.1	46.1	100%	0.011	100.00%	KJ942759.1
☑	Influenza A virus (A/reassortant/NYMC X-223(A/Puerto Rico/8/1934 x A/Texas/50/2012)(H3N	46.1	46.1	100%	0.011	100.00%	KJ942751.1
☑	Influenza A virus (A/reassortant/NYMC X-221(A/Puerto Rico/8/1934 x A/Ohio/2/2012)(H3N2)	46.1	46.1	100%	0.011	100.00%	KJ942743.1
☑	Influenza A virus (A/reassortant/NYMC X-179A(NYMC X-157 x A/California/07/2009)(H1N1))	46.1	46.1	100%	0.011	100.00%	KJ942735.1
☑	Influenza A virus (A/reassortant/NYMC X-215(A/Puerto Rico/8/1934 x A/Brisbane/299/2011)(	46.1	46.1	100%	0.011	100.00%	KJ942727.1
☑	Influenza A virus (A/reassortant/NYMC X-217(A/Puerto Rico/8/1934 x A/Victoria/361/2011)(H	46.1	46.1	100%	0.011	100.00%	KJ942719.1
☑	Influenza A virus (A/reassortant/NYMC X-217A(A/Puerto Rico/8/1934 x A/Victoria/361/2011)(	46.1	46.1	100%	0.011	100.00%	KJ942679.1
☑	Influenza A virus (A/reassortant/NYMC X-225(A/Puerto Rico/8/1934 x A/Hawaii/22/2012)(H3N	46.1	46.1	100%	0.011	100.00%	KJ942671.1
☑	Influenza A virus (A/reassortant/X-121(Puerto Rico/8/1934 x Shangdong/9/1993)(H3N2)) seg	46.1	46.1	100%	0.011	100.00%	KJ942663.1
☑	Influenza A virus (A/reassortant/NYMC X-203A(A/Puerto Rico/8/1934 x A/Minnesota/11/2010	46.1	46.1	100%	0.011	100.00%	KJ942647.1
☑	Influenza A virus (A/reassortant/NYMC X-179(NYMC X-157 x A/California/07/2009)(H1N1)) s	46.1	46.1	100%	0.011	100.00%	KJ942639.1
☑	Influenza A virus (A/reassortant/X-53(Puerto Rico/8/1934 x New Jersey/11/1976)(H1N1)) seg	46.1	46.1	100%	0.011	100.00%	KJ942607.1
☑	Influenza A virus (A/Boston/YGA_01016/2012(H3N2)) polymerase PB2 (PB2) gene, complete	46.1	46.1	100%	0.011	100.00%	CY168646.1
☑	Influenza A virus (A/duck/Jiangsu/1-MA/2008(H9N2)) segment 1 polymerase PB2 (PB2) gene	46.1	46.1	100%	0.011	100.00%	KF881737.1
☑	Influenza A virus (A/Puerto Rico/8/1934(H1N1)) polymerase PB2 (PB2) gene, complete cds	46.1	46.1	100%	0.011	100.00%	CY148250.1
☑	Influenza A virus (A/Puerto Rico/8-SV13/1934(H1N1)) polymerase PB2 (PB2) gene, complete	46.1	46.1	100%	0.011	100.00%	CY147533.1
☑	Influenza A virus (A/Puerto Rico/8-SV7/1934(H1N1)) polymerase PB2 (PB2) gene, complete	46.1	46.1	100%	0.011	100.00%	CY147493.1
☑	Influenza A virus (A/Hong Kong/JY2/1968(H3N2)) polymerase PB2 (PB2) gene, complete cds	46.1	46.1	100%	0.011	100.00%	CY147445.1
☑	Influenza A virus (A/New Jersey/Wistar/1976(H1N1)) polymerase PB2 (PB2) gene, complete	46.1	46.1	100%	0.011	100.00%	CY147429.1
☑	Influenza A virus (A/BH/JY2/1935(H1N1)) polymerase PB2 (PB2) gene, complete cds	46.1	46.1	100%	0.011	100.00%	CY147333.1
☑	Influenza A virus (A/Puerto Rico/8-SV120/1934(H1N1)) polymerase PB2 (PB2) gene, comple	46.1	46.1	100%	0.011	100.00%	CY146880.1
☑	Influenza A virus (A/Puerto Rico/8-SV20/1934(H1N1)) polymerase PB2 (PB2) gene, complete	46.1	46.1	100%	0.011	100.00%	CY146848.1
☑	Influenza A virus (A/Puerto Rico/8-LVD3/1934(H1N1)) polymerase PB2 (PB2) gene, complete	46.1	46.1	100%	0.011	100.00%	CY146840.1

Influenza K23

GGACAAACTTTATGGAGTAAAAT

Descriptions | Graphic Summary | Alignments | **Taxonomy**

Reports | **Lineage** | **Organism** | **Taxonomy**

100 sequences selected

Organism	Blast Name	Score	Number of Hits	Description
Influenza A virus	viruses	46.1	100	Influenza A virus hits

	Description	Max Score	Total Score	Query Cover	E value	Per. Ident	Accession
☑	Influenza A virus (A/garganey/North Kazakhstan/45/2018(H3N8)) segment 3 polymerase PA (	46.1	46.1	100%	0.011	100.00%	MT126634.1
☑	Influenza A virus (A/chicken/Viet Nam/HU12-668/2019(H5N1)) segment 3 polymerase PA (PA	46.1	46.1	100%	0.011	100.00%	MT106945.1
☑	Influenza A virus (A/chicken/Viet Nam/HU12-667/2019(H5N1)) segment 3 polymerase PA (PA	46.1	46.1	100%	0.011	100.00%	MT106937.1
☑	Influenza A virus (A/chicken/Viet Nam/HU12-666/2019(H5N1)) segment 3 polymerase PA (PA	46.1	46.1	100%	0.011	100.00%	MT106929.1
☑	Influenza A virus (A/chicken/Viet Nam/HU12-665/2019(H5N1)) segment 3 polymerase PA (PA	46.1	46.1	100%	0.011	100.00%	MT106921.1
☑	Influenza A virus (A/chicken/Viet Nam/HU12-669/2019(H5N1)) segment 3 polymerase PA (PA	46.1	46.1	100%	0.011	100.00%	MT106905.1
☑	Influenza A virus (A/Wisconsin/590/2019(H3N2)) segment 3 polymerase PA (PA) and PA-X pr	46.1	46.1	100%	0.011	100.00%	MT056609.1
☑	Influenza A virus (A/Wisconsin/589/2019(H3N2)) segment 3 polymerase PA (PA) and PA-X pr	46.1	46.1	100%	0.011	100.00%	MT056601.1
☑	Influenza A virus (A/Washington/669/2019(H3N2)) segment 3 polymerase PA (PA) and PA-X	46.1	46.1	100%	0.011	100.00%	MT056593.1
☑	Influenza A virus (A/Virginia/40/2019(H3N2)) segment 3 polymerase PA (PA) and PA-X protei	46.1	46.1	100%	0.011	100.00%	MT056585.1
☑	Influenza A virus (A/Texas/411/2019(H3N2)) segment 3 polymerase PA (PA) and PA-X protein	46.1	46.1	100%	0.011	100.00%	MT056561.1
☑	Influenza A virus (A/South Carolina/16/2019(H3N2)) segment 3 polymerase PA (PA) and PA-)	46.1	46.1	100%	0.011	100.00%	MT056553.1
☑	Influenza A virus (A/Pennsylvania/1036/2019(H3N2)) segment 3 polymerase PA (PA) and PA-	46.1	46.1	100%	0.011	100.00%	MT056545.1
☑	Influenza A virus (A/Pennsylvania/1028/2019(H3N2)) segment 3 polymerase PA (PA) and PA-	46.1	46.1	100%	0.011	100.00%	MT056537.1
☑	Influenza A virus (A/North Carolina/31/2019(H3N2)) segment 3 polymerase PA (PA) and PA-X	46.1	46.1	100%	0.011	100.00%	MT056529.1
☑	Influenza A virus (A/New York/61/2019(H3N2)) segment 3 polymerase PA (PA) and PA-X prot	46.1	46.1	100%	0.011	100.00%	MT056521.1
☑	Influenza A virus (A/New York/57/2019(H3N2)) segment 3 polymerase PA (PA) and PA-X prot	46.1	46.1	100%	0.011	100.00%	MT056513.1
☑	Influenza A virus (A/New York/54/2019(H3N2)) segment 3 polymerase PA (PA) and PA-X prot	46.1	46.1	100%	0.011	100.00%	MT056505.1
☑	Influenza A virus (A/New Jersey/43/2019(H3N2)) segment 3 polymerase PA (PA) and PA-X pr	46.1	46.1	100%	0.011	100.00%	MT056497.1
☑	Influenza A virus (A/New Jersey/41/2019(H3N2)) segment 3 polymerase PA (PA) and PA-X pr	46.1	46.1	100%	0.011	100.00%	MT056489.1
☑	Influenza A virus (A/New Hampshire/40/2019(H3N2)) segment 3 polymerase PA (PA) and PA-	46.1	46.1	100%	0.011	100.00%	MT056481.1
☑	Influenza A virus (A/New Hampshire/37/2019(H3N2)) segment 3 polymerase PA (PA) and PA-	46.1	46.1	100%	0.011	100.00%	MT056473.1
☑	Influenza A virus (A/Nevada/46/2019(H3N2)) segment 3 polymerase PA (PA) and PA-X protei	46.1	46.1	100%	0.011	100.00%	MT056465.1
☑	Influenza A virus (A/Nevada/46/2019(H3N2)) segment 3 polymerase PA (PA) and PA-X protei	46.1	46.1	100%	0.011	100.00%	MT056457.1
☑	Influenza A virus (A/Montana/68/2019(H3N2)) segment 3 polymerase PA (PA) and PA-X prote	46.1	46.1	100%	0.011	100.00%	MT056449.1
☑	Influenza A virus (A/Montana/68/2019(H3N2)) segment 3 polymerase PA (PA) and PA-X prote	46.1	46.1	100%	0.011	100.00%	MT056441.1
☑	Influenza A virus (A/Montana/66/2019(H3N2)) segment 3 polymerase PA (PA) and PA-X prote	46.1	46.1	100%	0.011	100.00%	MT056433.1
☑	Influenza A virus (A/Montana/66/2019(H3N2)) segment 3 polymerase PA (PA) and PA-X prote	46.1	46.1	100%	0.011	100.00%	MT056425.1
☑	Influenza A virus (A/Montana/65/2019(H3N2)) segment 3 polymerase PA (PA) and PA-X prote	46.1	46.1	100%	0.011	100.00%	MT056417.1
☑	Influenza A virus (A/Montana/65/2019(H3N2)) segment 3 polymerase PA (PA) and PA-X prote	46.1	46.1	100%	0.011	100.00%	MT056409.1
☑	Influenza A virus (A/Missouri/30/2019(H3N2)) segment 3 polymerase PA (PA) and PA-X prote	46.1	46.1	100%	0.011	100.00%	MT056401.1
☑	Influenza A virus (A/Mississippi/37/2019(H3N2)) segment 3 polymerase PA (PA) and PA-X pr	46.1	46.1	100%	0.011	100.00%	MT056393.1
☑	Influenza A virus (A/Massachusetts/43/2019(H3N2)) segment 3 polymerase PA (PA) and PA-)	46.1	46.1	100%	0.011	100.00%	MT056385.1
☑	Influenza A virus (A/Massachusetts/42/2019(H3N2)) segment 3 polymerase PA (PA) and PA-)	46.1	46.1	100%	0.011	100.00%	MT056377.1
☑	Influenza A virus (A/Maryland/41/2019(H3N2)) segment 3 polymerase PA (PA) and PA-X prot	46.1	46.1	100%	0.011	100.00%	MT056369.1
☑	Influenza A virus (A/Maryland/40/2019(H3N2)) segment 3 polymerase PA (PA) and PA-X prot	46.1	46.1	100%	0.011	100.00%	MT056361.1
☑	Influenza A virus (A/Maine/44/2019(H3N2)) segment 3 polymerase PA (PA) and PA-X protein	46.1	46.1	100%	0.011	100.00%	MT056353.1
☑	Influenza A virus (A/Maine/43/2019(H3N2)) segment 3 polymerase PA (PA) and PA-X protein	46.1	46.1	100%	0.011	100.00%	MT056345.1
☑	Influenza A virus (A/Maine/42/2019(H3N2)) segment 3 polymerase PA (PA) and PA-X protein	46.1	46.1	100%	0.011	100.00%	MT056337.1
☑	Influenza A virus (A/Maine/39/2019(H3N2)) segment 3 polymerase PA (PA) and PA-X protein	46.1	46.1	100%	0.011	100.00%	MT056329.1
☑	Influenza A virus (A/Louisiana/61/2019(H3N2)) segment 3 polymerase PA (PA) and PA-X prot	46.1	46.1	100%	0.011	100.00%	MT056321.1
☑	Influenza A virus (A/Louisiana/60/2019(H3N2)) segment 3 polymerase PA (PA) and PA-X prot	46.1	46.1	100%	0.011	100.00%	MT056313.1
☑	Influenza A virus (A/Idaho/47/2019(H3N2)) segment 3 polymerase PA (PA) and PA-X protein (	46.1	46.1	100%	0.011	100.00%	MT056305.1
☑	Influenza A virus (A/Idaho/47/2019(H3N2)) segment 3 polymerase PA (PA) and PA-X protein (	46.1	46.1	100%	0.011	100.00%	MT056297.1
☑	Influenza A virus (A/Hawaii/85/2019(H3N2)) segment 3 polymerase PA (PA) and PA-X protein	46.1	46.1	100%	0.011	100.00%	MT056289.1
☑	Influenza A virus (A/Hawaii/83/2019(H3N2)) segment 3 polymerase PA (PA) and PA-X protein	46.1	46.1	100%	0.011	100.00%	MT056281.1
☑	Influenza A virus (A/Florida/130/2019(H3N2)) segment 3 polymerase PA (PA) and PA-X prote	46.1	46.1	100%	0.011	100.00%	MT056273.1
☑	Influenza A virus (A/Florida/127/2019(H3N2)) segment 3 polymerase PA (PA) and PA-X prote	46.1	46.1	100%	0.011	100.00%	MT056265.1
☑	Influenza A virus (A/Delaware/58/2019(H3N2)) segment 3 polymerase PA (PA) and PA-X prot	46.1	46.1	100%	0.011	100.00%	MT056257.1
☑	Influenza A virus (A/Delaware/57/2019(H3N2)) segment 3 polymerase PA (PA) and PA-X prot	46.1	46.1	100%	0.011	100.00%	MT056249.1

KSHV K31

CTTTGGTCCGGGGTCCTTGCGTTGGATTTTT

Descriptions | Graphic Summary | Alignments | **Taxonomy**

Reports | **Lineage** | **Organism** | **Taxonomy**

44 sequences selected

Organism	Blast Name	Score	Number of Hits	Description
root			45	
. synthetic construct	other sequences	58.4	8	synthetic construct hits
. Human gammaherpesvirus 8	viruses	58.4	36	Human gammaherpesvirus 8 hits
. Human herpesvirus 8 type M	viruses	58.4	1	Human herpesvirus 8 type M hits

Description	Max Score	Total Score	Query Cover	E value	Per. Ident	Accession
Synthetic construct clone BAC16 K1 ITAM mutant sequence	58.4	58.4	100%	2e-06	100.00%	MK497257.1
Mutant Human gammaherpesvirus 8 clone BAC16_ORF21_3GVmutant, complete sequenc	58.4	58.4	100%	2e-06	100.00%	MN752405.1
Human gammaherpesvirus 8 clone BAC16, complete genome	58.4	58.4	100%	2e-06	100.00%	MK733609.1
Human gammaherpesvirus 8 strain UNC_KICS010, complete genome	58.4	58.4	100%	2e-06	100.00%	MK733608.1
Human gammaherpesvirus 8 strain BC1_UNC, complete genome	58.4	58.4	100%	2e-06	100.00%	MK733607.1
Human gammaherpesvirus 8 strain UNC_KICS009, complete genome	58.4	58.4	100%	2e-06	100.00%	MK733606.1
Synthetic construct clone BAC16 JSC-1, complete sequence	58.4	58.4	100%	2e-06	100.00%	MK208323.1
Human gammaherpesvirus 8 strain JSC-1, complete genome	58.4	58.4	100%	2e-06	100.00%	MK143395.1
Human gammaherpesvirus 8 DNA, nearly complete genome, strain: Japan1	58.4	58.4	100%	2e-06	100.00%	LC200589.1
Human gammaherpesvirus 8 DNA, nearly complete genome, strain: Miyako3	58.4	58.4	100%	2e-06	100.00%	LC200588.1
Human gammaherpesvirus 8 DNA, nearly complete genome, strain: Miyako2	58.4	58.4	100%	2e-06	100.00%	LC200587.1
Human gammaherpesvirus 8 DNA, nearly complete genome, strain: Miyako1	58.4	58.4	100%	2e-06	100.00%	LC200586.1
Synthetic construct clone BAC16-derived KSHV-IND, complete sequence	58.4	58.4	100%	2e-06	100.00%	KY246444.1
Synthetic construct clone BAC16-derived KSHV-LYT, complete sequence	58.4	58.4	100%	2e-06	100.00%	KY246443.1
Human herpesvirus 8 DNA, complete genome, strain: SPEL	58.4	58.4	100%	2e-06	100.00%	AP017458.1
Synthetic construct clone BAC16K1revertant genomic sequence	58.4	58.4	100%	2e-06	100.00%	KX189629.1
Synthetic construct clone BAC16K1flag genomic sequence	58.4	58.4	100%	2e-06	100.00%	KX189628.1
Synthetic construct clone BAC16K1_5xSTOP genomic sequence	58.4	58.4	100%	2e-06	100.00%	KX189627.1
Synthetic construct clone BAC16deltaK1 genomic sequence	58.4	58.4	100%	2e-06	100.00%	KX189626.1
Human herpesvirus 8 strain ZM130, partial genome	58.4	58.4	100%	2e-06	100.00%	KT271468.1
Human herpesvirus 8 strain ZM128, partial genome	58.4	58.4	100%	2e-06	100.00%	KT271467.1
Human herpesvirus 8 strain ZM124, partial genome	58.4	58.4	100%	2e-06	100.00%	KT271466.1
Human herpesvirus 8 strain ZM123, partial genome	58.4	58.4	100%	2e-06	100.00%	KT271465.1
Human herpesvirus 8 strain ZM121, partial genome	58.4	58.4	100%	2e-06	100.00%	KT271464.1
Human herpesvirus 8 strain ZM118, partial genome	58.4	58.4	100%	2e-06	100.00%	KT271463.1
Human herpesvirus 8 strain ZM117, partial genome	58.4	58.4	100%	2e-06	100.00%	KT271462.1
Human herpesvirus 8 strain ZM116, partial genome	58.4	58.4	100%	2e-06	100.00%	KT271461.1
Human herpesvirus 8 strain ZM114, partial genome	58.4	58.4	100%	2e-06	100.00%	KT271460.1
Human herpesvirus 8 strain ZM108, partial genome	58.4	58.4	100%	2e-06	100.00%	KT271459.1
Human herpesvirus 8 strain ZM106, partial genome	58.4	58.4	100%	2e-06	100.00%	KT271458.1
Human herpesvirus 8 strain ZM102, partial genome	58.4	58.4	100%	2e-06	100.00%	KT271457.1
Human herpesvirus 8 strain ZM095, partial genome	58.4	58.4	100%	2e-06	100.00%	KT271456.1
Human herpesvirus 8 strain ZM091, partial genome	58.4	58.4	100%	2e-06	100.00%	KT271455.1
Human herpesvirus 8 strain ZM027, partial genome	58.4	58.4	100%	2e-06	100.00%	KT271454.1
Human herpesvirus 8 strain ZM004, partial genome	58.4	58.4	100%	2e-06	100.00%	KT271453.1
Human herpesvirus 8 clone BrK.219#1_4, complete genome	58.4	58.4	100%	2e-06	100.00%	KF588566.1
Human herpesvirus 8 strain DG1, complete genome	58.4	58.4	100%	2e-06	100.00%	JQ619843.1
Human herpesvirus 8 isolate KSHV-BAC36-deltaK15, partial genome	58.4	58.4	100%	2e-06	100.00%	JX228174.1
Human herpesvirus 8 strain KSHV-BAC36 long unique region, genomic sequence	58.4	58.4	100%	2e-06	100.00%	HQ404500.1
Human herpesvirus 8 strain JSC-1 clone BAC16, complete genome	58.4	58.4	100%	2e-06	100.00%	GQ994935.1
Kaposi's sarcoma-associated herpesvirus glycoprotein M, DNA replication protein, glycopro	58.4	58.4	100%	2e-06	100.00%	U93872.2
Human herpesvirus 8 strain GK18, complete genome	58.4	58.4	100%	2e-06	100.00%	NC_009333.1
Human herpesvirus 8 type M, partial genome	58.4	58.4	100%	2e-06	100.00%	U75698.1
Kaposi's sarcoma-associated herpes-like virus clone KS5, ORF20-ORF35 gene region	58.4	58.4	100%	2e-06	100.00%	U40377.1

KSHV K31

AACAAGCAGACACACGCCCCAGTACACATGG

Descriptions | Graphic Summary | Alignments | **Taxonomy**

Reports | **Lineage** | **Organism** | **Taxonomy**

47 sequences selected

Organism	Blast Name	Score	Number of Hits	Description
root			48	
. synthetic construct	other sequences	58.4	8	synthetic construct hits
. Human gammaherpesvirus 8	viruses	58.4	37	Human gammaherpesvirus 8 hits
. Human herpesvirus 8 type M	viruses	58.4	1	Human herpesvirus 8 type M hits
. Equid gammaherpesvirus 2	viruses	38.1	2	Equid gammaherpesvirus 2 hits

Description	Max Score	Total Score	Query Cover	E value	Per. Ident	Accession
Synthetic construct clone BAC16 K1 ITAM mutant sequence	58.4	58.4	100%	2e-06	100.00%	MK497257.1
Mutant Human gammaherpesvirus 8 clone BAC16_ORF21_3GVmutant, complete sequenc	58.4	58.4	100%	2e-06	100.00%	MN752405.1
Human gammaherpesvirus 8 clone BAC16, complete genome	58.4	58.4	100%	2e-06	100.00%	MK733609.1
Human gammaherpesvirus 8 strain UNC_KICS010, complete genome	58.4	58.4	100%	2e-06	100.00%	MK733608.1
Human gammaherpesvirus 8 strain BC1_UNC, complete genome	58.4	58.4	100%	2e-06	100.00%	MK733607.1
Human gammaherpesvirus 8 strain UNC_KICS009, complete genome	58.4	58.4	100%	2e-06	100.00%	MK733606.1
Synthetic construct clone BAC16 JSC-1, complete sequence	58.4	58.4	100%	2e-06	100.00%	MK208323.1
Human gammaherpesvirus 8 strain JSC-1, complete genome	58.4	58.4	100%	2e-06	100.00%	MK143395.1
Human gammaherpesvirus 8 DNA, nearly complete genome, strain: Japan1	58.4	58.4	100%	2e-06	100.00%	LC200589.1
Synthetic construct clone BAC16-derived KSHV-IND, complete sequence	58.4	58.4	100%	2e-06	100.00%	KY246444.1
Synthetic construct clone BAC16-derived KSHV-LYT, complete sequence	58.4	58.4	100%	2e-06	100.00%	KY246443.1
Human herpesvirus 8 DNA, complete genome, strain: SPEL	58.4	58.4	100%	2e-06	100.00%	AP017458.1
Synthetic construct clone BAC16K1revertant genomic sequence	58.4	58.4	100%	2e-06	100.00%	KX189629.1
Synthetic construct clone BAC16K1flag genomic sequence	58.4	58.4	100%	2e-06	100.00%	KX189628.1
Synthetic construct clone BAC16K1_5xSTOP genomic sequence	58.4	58.4	100%	2e-06	100.00%	KX189627.1
Synthetic construct clone BAC16deltaK1 genomic sequence	58.4	58.4	100%	2e-06	100.00%	KX189626.1
Human herpesvirus 8 strain ZM130, partial genome	58.4	58.4	100%	2e-06	100.00%	KT271468.1
Human herpesvirus 8 strain ZM128, partial genome	58.4	58.4	100%	2e-06	100.00%	KT271467.1
Human herpesvirus 8 strain ZM124, partial genome	58.4	58.4	100%	2e-06	100.00%	KT271466.1
Human herpesvirus 8 strain ZM123, partial genome	58.4	58.4	100%	2e-06	100.00%	KT271465.1
Human herpesvirus 8 strain ZM121, partial genome	58.4	58.4	100%	2e-06	100.00%	KT271464.1
Human herpesvirus 8 strain ZM117, partial genome	58.4	58.4	100%	2e-06	100.00%	KT271462.1
Human herpesvirus 8 strain ZM116, partial genome	58.4	58.4	100%	2e-06	100.00%	KT271461.1
Human herpesvirus 8 strain ZM114, partial genome	58.4	58.4	100%	2e-06	100.00%	KT271460.1
Human herpesvirus 8 strain ZM108, partial genome	58.4	58.4	100%	2e-06	100.00%	KT271459.1
Human herpesvirus 8 strain ZM106, partial genome	58.4	58.4	100%	2e-06	100.00%	KT271458.1
Human herpesvirus 8 strain ZM102, partial genome	58.4	58.4	100%	2e-06	100.00%	KT271457.1
Human herpesvirus 8 strain ZM095, partial genome	58.4	58.4	100%	2e-06	100.00%	KT271456.1
Human herpesvirus 8 strain ZM091, partial genome	58.4	58.4	100%	2e-06	100.00%	KT271455.1
Human herpesvirus 8 strain ZM027, partial genome	58.4	58.4	100%	2e-06	100.00%	KT271454.1
Human herpesvirus 8 strain ZM004, partial genome	58.4	58.4	100%	2e-06	100.00%	KT271453.1
Human herpesvirus 8 clone BrK.219#1_4, complete genome	58.4	58.4	100%	2e-06	100.00%	KF588566.1
Human herpesvirus 8 strain DG1, complete genome	58.4	58.4	100%	2e-06	100.00%	JQ619843.1
Human herpesvirus 8 isolate KSHV-BAC36-deltaK15, partial genome	58.4	58.4	100%	2e-06	100.00%	JX228174.1
Human herpesvirus 8 strain KSHV-BAC36 long unique region, genomic sequence	58.4	58.4	100%	2e-06	100.00%	HQ404500.1
Human herpesvirus 8 strain JSC-1 clone BAC16, complete genome	58.4	58.4	100%	2e-06	100.00%	GQ994935.1
Kaposi's sarcoma-associated herpesvirus glycoprotein M, DNA replication protein, glycopro	58.4	58.4	100%	2e-06	100.00%	U93872.2
Human herpesvirus 8 strain GK18, complete genome	58.4	58.4	100%	2e-06	100.00%	NC_009333.1
Human herpesvirus 8 type M, partial genome	58.4	58.4	100%	2e-06	100.00%	U75698.1
Kaposi's sarcoma-associated herpes-like virus clone KS5, ORF20-ORF35 gene region	58.4	58.4	100%	2e-06	100.00%	U40377.1
Kaposi's sarcoma-associated herpes-like virus putative minor capsid protein gene, complete	58.4	58.4	100%	2e-06	100.00%	U18551.1
Human gammaherpesvirus 8 DNA, nearly complete genome, strain: Miyako3	52.8	52.8	100%	1e-04	96.77%	LC200588.1
Human gammaherpesvirus 8 DNA, nearly complete genome, strain: Miyako2	52.8	52.8	100%	1e-04	96.77%	LC200587.1
Human gammaherpesvirus 8 DNA, nearly complete genome, strain: Miyako1	52.8	52.8	100%	1e-04	96.77%	LC200586.1
Human herpesvirus 8 strain ZM118, partial genome	52.8	52.8	100%	1e-04	96.77%	KT271463.1
Equid herpesvirus 2 strain G9/92, complete genome	38.1	38.1	83%	2.8	92.31%	KM924294.1
Equid herpesvirus 2 strain 86/67, complete genome	38.1	38.1	83%	2.8	92.31%	U20824.2
