# Supplementary material for: KITSUNE: A Tool for Identifying Empirically Optimal K-mer Length for Alignment-Free Phylogenomic Analysis
Source: Front Bioeng Biotechnol. 2020 Sep 23;8:556413. doi: 10.3389/fbioe.2020.556413 (PMC7538862; doi:10.3389/fbioe.2020.556413)
Supplement: Supplementary file 2 [file Data_Sheet_2.PDF]

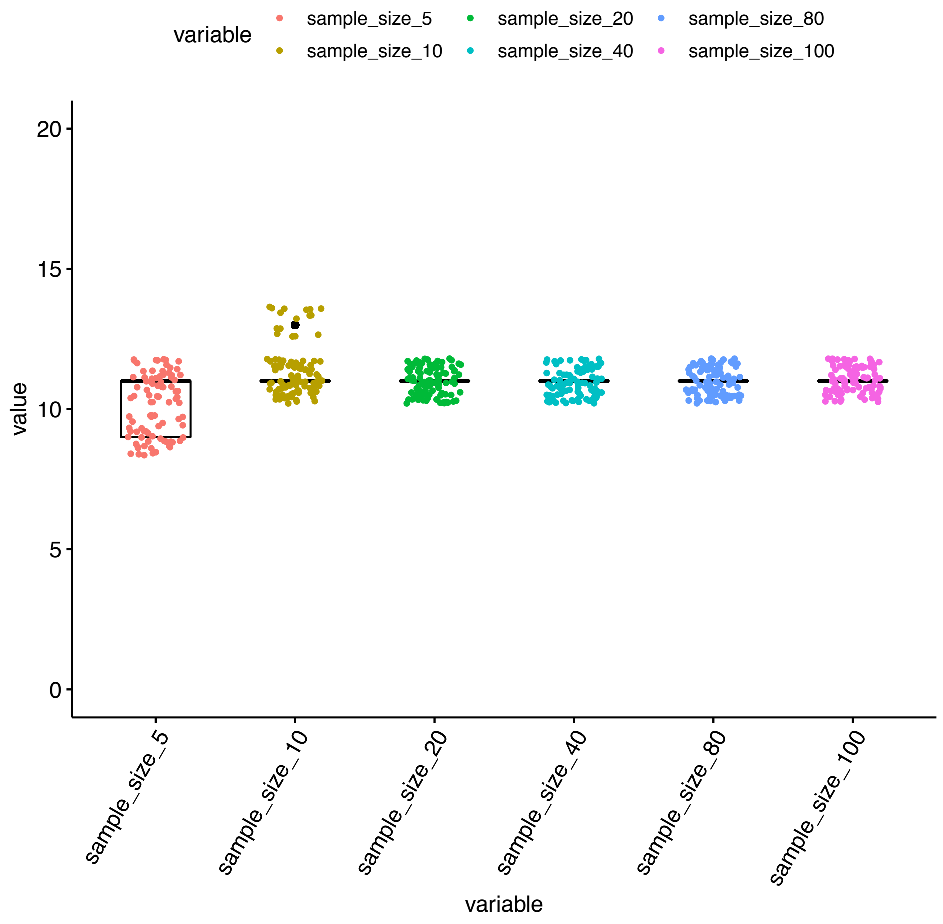

Figure S1 Impact of subsampling size on optimal k-mer length identification for the virus data.

A)

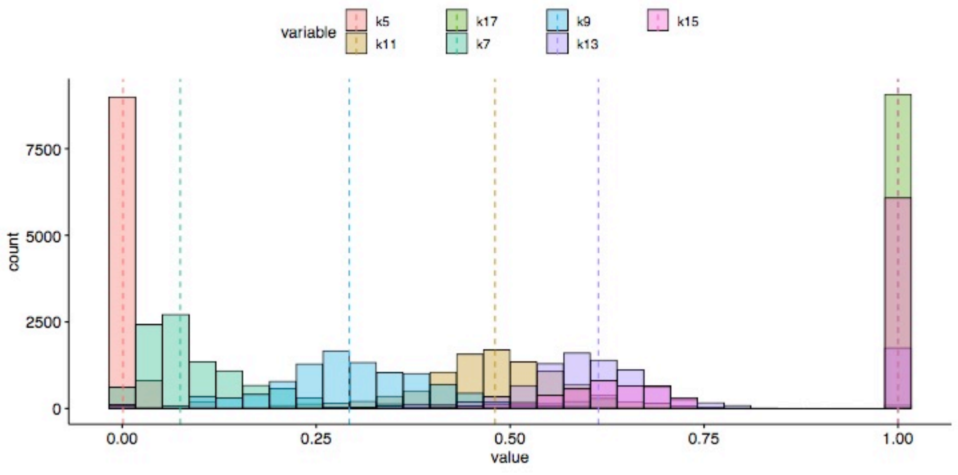

B)

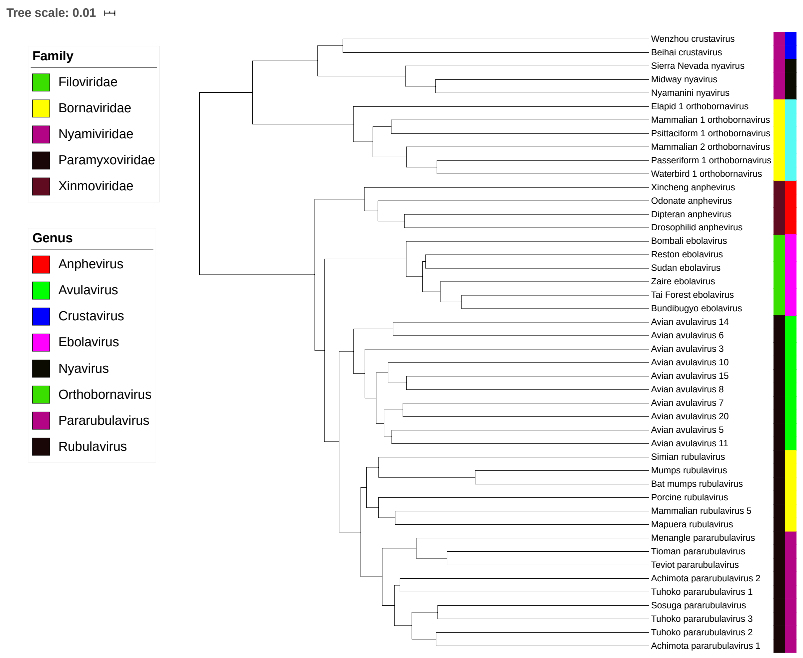

Figure S2 A) Histograms with rug plots show the distributions of genomic distances (transformed Jaccard) base on the identified optimal k-mer length (k11) with the other. B) A tree show a good discrimination of different virus family and genus by using using transformed Jaccard based on the identified optimum k-mer length (k11).

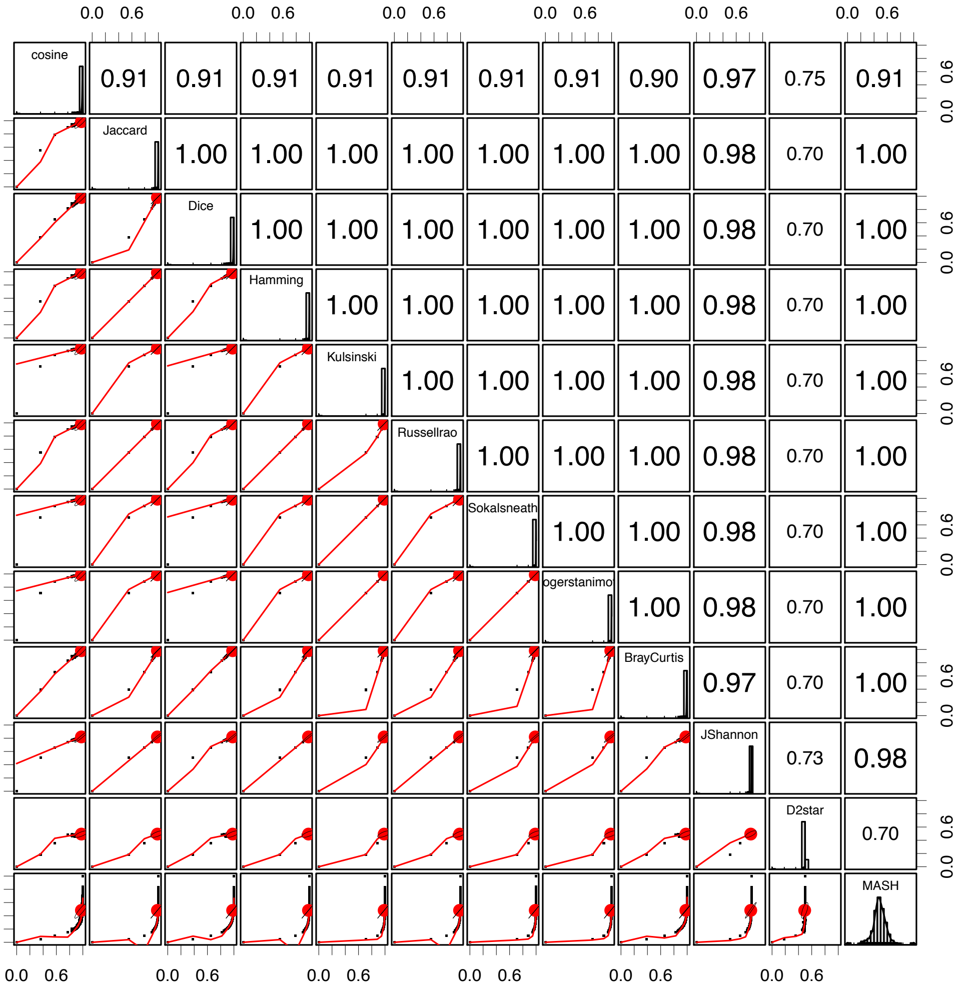

Figure S3. Correlogram plot comparing genomic distances calculated with different methods based on 100 viral genomes. Lower left triangle: scatter plots of genomic distances derived from of pairs of genomes calculated with different methods with correlation ellipses; upper right triangle: Spearman's rank correlation coefficients for different methods; diagonal boxes: distribution of genomic distances.
